# Triage and care for women with symptoms or diagnosis of pregnancy loss between 14 + 0 and 21 + 6 weeks' gestation

**DOI:** 10.1002/ijgo.70621

**Published:** 2025-11-24

**Authors:** Caroline E. Fox, Rosinder Kaur, Kugajeevan Vigneswaran, Rachel Small, Jenny Carter, Keelin O'Donoghue, Alexander E. P. Heazell, Anna L. David, Nigel Simpson, Angharad Care, Lisa Starrs, Andrew Shennan, Catalina María Valencia González, Priya Soma‐Pillay, Leah Fitzsimmons, Adam J. Devall, Arri Coomarasamy, Mid‐trimester Pregnancy Loss Guideline Consensus Panel, Owen Arthurs, Ruth Bender‐Atik, Phil Bennett, Kirstien Boelaert, Elizabeth Bonney, Leanna Brace, Naomi Carlisle, Davide Casagrandi, Manju Chandiramani, Carolyn Chiswick, Alexandra Emms, Stella Fielder, James Goadsby, Laura Goodfellow, Jim Gray, Jemma Johns, Emma Kirk, Will Lester, Sophie Mackay, Maria Memtsa, Katie Morris, Sunbal Mukhtar, Naimah Raza, Rita Sarquis, Neil Sebire, Lucy Smith, Lucy Spencer, Amos Tetteh, Natalie Woodhead

**Affiliations:** ^1^ Birmingham Women's and Hospital Birmingham Women's and Children's NHS Foundation Trust Birmingham UK; ^2^ Tommy's National Centre for Miscarriage Research Birmingham UK; ^3^ School of Medical Sciences University of Birmingham Birmingham UK; ^4^ King's Fertility London UK; ^5^ Birmingham Heartlands Hospital University Hospitals Birmingham NHS Foundation Trust Birmingham UK; ^6^ Department of Women and Children's Health King's College London London UK; ^7^ Tommy's National Centre for Preterm Birth Research London UK; ^8^ Pregnancy Loss Research Group University College Cork Cork Ireland; ^9^ University of Manchester Manchester UK; ^10^ Tommy's Stillbirth Research Centre University of Manchester Manchester UK; ^11^ Elizabeth Garrett Anderson Institute for Women's Health University College London London UK; ^12^ National Institute for Health and Care Research University College London Hospitals Biomedical Research Centre London UK; ^13^ Leeds Institute of Medical Research University of Leeds Leeds UK; ^14^ Liverpool Women's Hospital Liverpool Women's NHS Foundation Trust Liverpool UK; ^15^ Department of Women and Children's Health University of Liverpool Liverpool UK; ^16^ Royal Infirmary of Edinburgh, NHS Lothian Edinburgh Scotland, UK; ^17^ Universidad Colegio Mayor de Nuestra Señora del Rosario Bogota Colombia; ^18^ University of Pretoria Pretoria South Africa

**Keywords:** diagnosis and management, miscarriage, pregnancy loss, second‐trimester loss, triage

## Abstract

Mid‐trimester pregnancy loss (MTL), defined as a pregnancy loss occurring between 14 + 0 and 21 + 6 weeks of gestation, causes significant physical and emotional distress to women and presents clinical challenges to healthcare professionals. It is acknowledged that in low‐resource settings, this guideline might be applicable to births up to 28 weeks or babies weighing less than 1 kg. Risk factors for MTL include advanced maternal age, previous history of MTL, women of Black ethnicity, smoking, excessive alcohol consumption, obesity, and anatomical factors such as a short cervix, congenital uterine anomalies, and myomas. Medical risk factors include previous cervical trauma from loop electrosurgical excision procedure or Cesarean section in labor, placental dysfunction, infections, thrombophilias, endocrine disorders such as thyroid disease and polycystic ovary syndrome, and fetal chromosomal abnormalities. Early assessment and accurate diagnosis are fundamental to managing threatened and confirmed mid‐trimester pregnancy loss. Our guideline emphasizes the importance of maternal vital signs monitoring, laboratory investigations, and ultrasound imaging to identify and manage those with threatened or confirmed mid‐trimester pregnancy loss, as well as address potential maternal complications, including infection or hemorrhage. A multidisciplinary approach involving obstetricians, gynecologists, maternal‐fetal medicine specialists, nurses, midwives, psychologists, and social workers is important for providing comprehensive care. The guideline advocates for personalized management plans tailored to individual women's preferences, medical history, and gestational age. Care for threatened MTL should be targeted to the likely cause and might include cervical cerclage, progesterone, and management of risk factors, for example antibiotics for urinary tract infections. Care for confirmed MTL might include expectant management, medical induction of labor, or surgical intervention such as dilation and evacuation. Acknowledging the profound emotional impact of mid‐trimester pregnancy loss, our guideline underscores the importance of offering compassionate and culturally sensitive psychosocial support to women and their families. This includes providing access to bereavement care, counseling services, support groups, and resources for coping with grief and loss. Continued monitoring and follow‐up care are essential components of managing mid‐trimester pregnancy loss. Our guideline recommends regular postpartum assessments to evaluate physical recovery and emotional well‐being and to address any ongoing medical or psychological concerns. Contraceptive counseling and future pregnancy planning should also be discussed as part of comprehensive care. It is important that, where possible, women receive continuity of care from healthcare professionals to help the coordination and provision of holistic and comprehensive care. Further research is needed to enhance our understanding of the etiology, risk factors, and optimal management strategies for threatened mid‐trimester pregnancy loss. Additionally, education and training initiatives should be implemented to ensure healthcare professionals are equipped with the knowledge and skills necessary to deliver high‐quality, woman‐centered care to individuals and families experiencing this complication. Mid‐trimester pregnancy loss represents a complex clinical scenario necessitating a holistic and compassionate approach to care. By adhering to the recommendations outlined in this clinical guideline, healthcare providers can strive to optimize outcomes and support individuals and their families through this challenging experience.

## 1 PURPOSE AND SCOPE

This document serves as a guide for the optimal triage and care of women presenting with symptoms of pregnancy loss between 14 + 0 and 21 + 6 weeks, referred to as mid‐trimester pregnancy loss (MTL) throughout this guideline. This guideline is intended to assist clinical judgment and not to replace it. Although it is not the focus of this guideline, our recommendations might also be appropriate in low‐resource settings for pregnancy losses between 22 and 28 weeks.

In this guideline, we use the terms “woman” or “women” throughout. These should be taken to include people who do not necessarily identify as women but are pregnant or have given birth.

## 2 TARGET AUDIENCE

This guidance is intended for the management of symptomatic women, as asymptomatic women with known risk factors should already be identified at pregnancy booking and offered specialist preterm prevention care.

This document is directed at multiple stakeholders with the intention of bringing attention to MTL. This document proposes to create a global framework for action to improve the diagnosis and care of women with symptoms or a diagnosis of MTL.

The intended target audience includes:

- Healthcare professionals—all those who are qualified to care for pregnant women and their babies (e.g., obstetricians, gynecologists, neonatologists, general practitioners, midwives, nurses, non‐physician clinicians, and clinical officers).
- Healthcare delivery organizations and providers—governments, federal and state legislators, healthcare management organizations, health insurance organizations, international development agencies, and nongovernmental organizations.
- Professional organizations—international, regional, and national professional organizations of obstetricians and gynecologists, midwives, nurses and neonatologists, and worldwide national organizations dedicated to the care of pregnant women.

## 3 IDENTIFICATION AND ASSESSMENT OF EVIDENCE

The Cochrane Library (including the Cochrane Database of Systematic Reviews), EMBASE, TRIP, Medline, and PubMed (electronic databases) were searched for relevant studies. The date of the last search was August 2025.

The databases were searched using the relevant MeSH terms including all subheadings. The search terms included “late abortion”, “late miscarriage”, “mid‐trimester/midtrimester miscarriage” and “second trimester miscarriage”. The search was limited to humans and the English language. The National Library for Health and National Guidelines Clearing House were also searched for relevant guidelines and reviews. The full search strategy is available to view as supporting information.

In assessing the quality of evidence and grading of strength of recommendations, the document follows the terminology proposed by the Grading of Recommendations, Assessment, Development and Evaluation (GRADE) Working Group (www.gradeworkinggroup.org/index.htm).

This system uses consistent language and graphical descriptions for the strength and quality of the recommendations and the evidence on which they are based. The overall quality of evidence was assessed for each of the recommendations and expressed using four levels of quality: very low, low, moderate, and high.

Considerations for quality of evidence include primarily the study design and methodology. This utilized the appropriate hierarchy of evidence as relevant to the study type (e.g., diagnostic, intervention/therapy, or prognostic). However, other factors considered in assessing the level of evidence were: risk of bias, study limitations, directness, consistency of results, precision, publication bias, indirectness of evidence, and scarcity of evidence.

Each recommendation is denoted with its strength (strong or weak) while considering the balance of desirable and undesirable consequences, quality of evidence, values and preferences, and resource use. Therefore, the quality of evidence is only one possible consideration for the strength of evidence. The decision to apply a possible examination or intervention is also based on potential risk–benefit, cost, and resource allocation. Some recommendations might be based on low‐quality evidence but still represent a benefit that outweighs the risks and burdens and therefore might be strongly recommended.

These recommendations are based on published evidence. However, given the limitations in the evidence, a consensus approach was needed to formulate many of the recommendations. The consensus group was multidisciplinary and met both face to face and virtually from 2022. Charity partners included Tommy's and the Miscarriage Association. We also thank our patient experts for their input as well.

## 4 MID‐TRIMESTER PREGNANCY LOSS: BACKGROUND, DEFINITION, RISK FACTORS, RISKS, AND PATHOPHYSIOLOGY

### 4.1 Introduction

Mid‐trimester pregnancy loss (MTL) is generally defined as a pregnancy loss between 14‐ and 24‐weeks' gestation.^1^ The terminology and definitions used in the published literature in relation to MTL, however, vary considerably. When referring to any pregnancy loss, the term “abortion” should be avoided as it can cause distress at an already difficult time. Qualitative work into the use of appropriate terminology has shown that parents felt “mid‐trimester pregnancy loss” as opposed to “mid‐trimester miscarriage” or “late miscarriage,” best represented their experience, and therefore is the term recommended for use in this guideline.^2^ For consistency, therefore, where previous studies have used the term miscarriage but not defined which trimester this relates to, this is referred to as early pregnancy loss.

The true incidence of MTL is difficult to ascertain; however, it is estimated to affect 2%–3% of recognized pregnancies.^3^ In published literature, MTL has often been merged with first trimester pregnancy losses, preterm birth (PTB), and preterm pre‐labor rupture of membranes (PPROM). These conditions might represent a continuum with shared underlying etiologies.

The distinction between MTL and PTB remains at the gestation thought to represent fetal viability (currently 24 weeks' gestation). It should be noted, however, that with advances in neonatal care management, survival‐focused care can be offered from 22 weeks' gestation depending on available resources, and this is why this guidance uses 21 + 6 as its cut‐off.^4^ It is acknowledged that in low‐resource settings, this guideline might be applicable to births up to 28 weeks or babies weighing less than 1 kg.

### 4.2 Risk factors for mid‐trimester pregnancy loss

Women with a history of MTL represent a heterogeneous group, demonstrating a wide range of presenting symptoms as well as etiologies. Contributing factors might include cervical insufficiency, infection, thrombophilia, and congenital uterine anomalies.

Although an underlying causative factor can be identified in approximately 50% of women with MTL, it remains unknown for a significant proportion of cases.^5^ Additionally, there might also be more than one contributing factor toward an MTL. The presence of dual or even triple pathology is thought to dramatically increase the risk of subsequent MTL.

When reviewing the evidence for MTL risk factors, we prioritized studies involving unselected cohorts or naturally conceived pregnancies to ensure findings are broadly applicable to the general obstetric population. However, several available studies were derived from in vitro fertilization (IVF) cohorts, and as such might differ in baseline characteristics, such as maternal age, comorbidities as well an underlying infertility diagnosis. Consequently, the generalizability of findings from IVF population studies to spontaneously conceived pregnancies is limited, and we acknowledge that such studies should be interpreted with caution in the context of wider clinical practice. There is heterogenity in the reporting of study outcomes. Where possible, we have reported the adjusted estimates.

#### 4.2.1 Epidemiological risk factors

##### 4.2.1.1 Maternal age

Advancing maternal age has been associated with an overall increase in the risk of early pregnancy loss. Sporadic early pregnancy loss related to embryo aneuploidy occurs most commonly in the first trimester and is, therefore, not strongly implicated in MTL. Nonetheless, maternal age is still considered a risk factor for MTL.

In a large Canadian population‐based study of 1040 miscarriages between 15 and 21 weeks' gestation, wherein fetal chromosomal or structural abnormalities were excluded, maternal age appeared to show a U‐shaped relationship with MTL risk.^3^ Using age 27 as the reference (odds ratio [OR] = 1.0), the odds of loss were elevated in both the youngest (≤16 years: OR = 3.0) (95% confidence interval [CI] 0.3–1.6) and oldest (≥44 years: OR = 8.9) (95% CI 1.1–4.9) mothers. Risk declined from the age of 16 to mid‐20s, reaching its lowest point at 27 years, then rose progressively from the late 20s onward, with a marked increase after age 35. The authors concluded that both very young ages as well as advanced maternal age were independent risk factors for MTL.

A large retrospective single‐center study, of 15 210 IVF‐conceived pregnancies found that women aged between 35 and 40 years were at a higher risk of late miscarriage, compared to women under 35, with an OR of 1.16 (95% CI 0.87–1.55). Women aged above 40 years had a further increased risk of late miscarriage, compared to women under 35 with an OR of 2.79 (95% CI 1.64–4.77).^6^ Importantly, patients who had pre‐implantation genetic testing were excluded, thereby adding to the generalisability of the findings to an unselected or natural conception population.

##### 4.2.1.2 Previous history of mid‐trimester loss

A history of MTL has been associated with an increased risk of recurrence in future pregnancies. The estimated overall recurrence risk of MTL is thought to be 7% based on birth registry data.^7^ If there is a known cause detected for the first MTL, several studies have found that a woman's risk of a recurrent MTL can be further stratified depending on the underlying phenotype of the initial MTL, with studies showing rates up to 30%.

Danish birth registry data followed 9602 women with either an MTL or extreme preterm birth (16 + 0 to 27 + 6 gestational weeks) in their first pregnancy. The overall recurrence rate was 7.3% (n = 452), a rate that differed by phenotype from <5% (fetal anomaly, multiple gestations, and intrauterine fetal death) to 21% (cervical insufficiency).^7^

A similar variation in recurrence risk by phenotype was seen in a single‐center cohort of 1072 IVF patients with an index MTL. Subsequent pregnancy outcomes were compared across four groups defined by the attributed cause of the index MTL: unexplained (*n* = 458), fetal (*n* = 146), cervical (*n* = 412), and trauma related (*n* = 56). Although “cervical” and “trauma related” were not defined in detail, the risk of another MTL in the next pregnancy was higher when the index loss was unexplained (9.4%) or cervical (15.5%) than when it was due to fetal or trauma‐related causes (4.2%): unexplained versus fetal/trauma, adjusted OR 1.9 (95% CI 1.2–2.9), *P* = 0.003; cervical versus fetal/trauma, adjusted OR 2.7 (95% CI 1.8–4.0), *P* < 0.001.^8^

Future risk of MTL might also be influenced by a shortened interpregnancy interval. One retrospective cohort study found that an interpregnancy interval (IPI) of ≤3 months was associated with an increased risk of recurrent MTL. A total of 4290 women who naturally conceived within 2 years following a loss at 14–23 weeks' gestation (miscarriage, termination, or perinatal death) were followed up to determine the risk of MTL in a subsequent pregnancy. The findings showed that following the loss of an index pregnancy of 14–19 weeks' gestation, an IPI of ≤3 months was associated with an increased rate of recurrent MTL compared with an IPI of >9–12 months (adjusted relative risk [aRR] = 2.02, 95% CI 1.44–2.83). This elevated risk was not observed for women who had a spontaneous index loss in the first trimester or between 20 and 23 weeks.^9^

##### 4.2.1.3 Ethnicity

There is a paucity of data available on the relationship between ethnicity and overall risk of early pregnancy loss, including specifically the risk of MTL.

One large retrospective study undertaken in the UK found that compared to white Europeans, the odds of a spontaneous miscarriage were increased in Black African (adjusted OR [aOR] 1.20; 95% CI 1.12–1.29) and Black Caribbean women (aOR 1.31; 95% CI 1.21–1.41). A previous history of spontaneous miscarriage was associated with preterm birth in all races but was strongest in Black African women (aOR 1.47; 95% CI 1.29–1.67), which led to the authors calling for further study of risk factors, specifically related to ethnicity.^10^

A prospective cohort study of 4070 women, conducted in the USA, studied MTL risk according to ethnicity and found that self‐reported race was independently associated with risk of early pregnancy loss, and the higher risk for Black women (*n* = 932) was concentrated in gestational weeks 10–20.^11^ Overall, the cumulative risk of pregnancy loss after 5 weeks' gestation was 21.3%. Black women had an increased risk of early pregnancy loss, after adjustment for age and alcohol use, compared to white women (adjusted hazard ratio = 1.57, 95% CI 1.27–1.93). When only pregnancy loss after 10 weeks gestation was considered, there was a further increase in the risk difference for Black women (adjusted hazard ratio = 1.93, 95% CI 1.48–2.51).

##### 4.2.1.4 Smoking

Smoking has been shown to increase the overall risk of spontaneous pregnancy loss,^16^ and a high‐quality meta‐analysis of studies from first trimester data shows that active smoking specifically during the pregnancy in which miscarriage risk was documented increases overall miscarriage risk (summary relative risk ratio = 1.32, 95% CI 1.21, 1.44; *n* = 25 studies). The risk of miscarriage increased with the amount smoked (1% increase in relative risk per cigarette smoked per day).^12^

Evidence isolating smoking‐related risk for MTL is limited and mixed. In a Danish prospective cohort of 24 608 pregnancies with exposure recorded before outcomes, maternal smoking was not associated with either first‐trimester loss (*n* = 104) or second‐trimester loss (*n* = 217) after adjustment.^13^ By contrast, a US case–control study (626 cases, 1300 controls; miscarriage endpoint ≤20 weeks) reported a stronger association for later losses, with moderate active smoking (11–20 cigarettes/day) being linked to MTL (crude OR 1.8; 95% CI 1.1–2.9), attenuating after adjustment (OR 1.5; 95% CI 0.9–2.6; model without environmental tobacco smoke). Environmental tobacco smoke exposure of ≥1 h/day was more strongly related to late than early losses (adjusted OR 1.9 vs 1.3).^14^

In relation to MTL, one study using outcomes from a Norwegian birth registry appeared to show that in utero exposure to tobacco smoke associated with a higher risk of MTL.^15^ The adjusted hazard ratio of late miscarriage was 1.23 (95% CI 0.72–2.12) in women with in utero exposure to maternal tobacco smoke when compared with non‐exposed women.

##### 4.2.1.5 Alcohol consumption

Heavy alcohol consumption is associated with an increased risk of MTL and early sPTB (spontaneous preterm birth). The threshold of risk is not easily defined but there does appear to be a dose‐dependent relationship.

In a dose–response meta‐analysis, the association between alcohol consumption and binge drinking and the risk of pregnancy loss in the first and second trimesters was evaluated. Adjusted data from 458 154 women in the second trimester analysis showed the risk of MTL increased by 3% (OR 1.03, 95% CI 0.99–1.08) for each additional drink per week but not to a statistically significant degree.^16^ However, the study that carried the greatest weight within this meta‐analysis found there was an increased risk of MTL for women consuming three or more alcoholic drinks daily, independent of age, parity, race, marital status, smoking, or previous abortions.^17^ Life‐table analysis showed that the age‐adjusted relative risks of second‐trimester losses (15–27 weeks) were 1.03 (not significant), 1.98 (*P <* 0.01), and 3.53 (*P <* 0.01) for women taking less than 1, 1–2, and 3 or more drinks daily, compared with non‐drinkers.^17^

##### 4.2.1.6 Obesity

Obesity has become a major health problem worldwide and is also associated with adverse pregnancy outcomes. Evidence examining the association between obesity and MTL is limited, as most studies report miscarriage risk across all gestations or focus on preterm birth outcomes. One prospective population‐based cohort study including 3604 women showed that obesity in women was associated with increased odds of miscarriage, independent of fertility treatment. Compared with normal weight in women, being overweight (body mass index [BMI] 25.0–29.9; OR 1.49; 95% CI 1.12–1.98) as well as obesity (≥30.0) (OR 1.44; 95% CI 1.00–2.08) were associated with increased odds of miscarriage.^18^

In relation to MTL risk, one study examining risk following an IVF cycle showed that obesity, defined as a BMI of 30 kg/m^2^ or above, was associated with a higher risk of MTL, with an OR of 1.80 (95% CI 1.33–2.44), compared to BMI between 18.5 and 25 kg/m^2^.^6^

##### 4.2.1.7 Environmental factors

The association between environmental risk factors and MTL is mainly extrapolated from studies that have examined the potential role of environmental exposures in increasing the risk of early pregnancy loss. These studies have generally focused on factors like air pollution, chemicals, and heavy metals. A systematic review including 35 human studies suggested that environmental factors such as air pollution might increase the risk of both spontaneous early pregnancy loss as well as still birth.^19^ Extrapolation of these findings could imply that MTL risk might also be elevated. However, these results are limited by several factors such as difficulties in controlling for confounding factors, reporting of data on degree of exposure and measurements of toxin dose.

#### 4.2.2 Anatomical factors

##### 4.2.2.1 Cervical integrity

Cervical insufficiency is a well‐established risk factor for MTL.

As there is currently no reliable test that can identify women with cervical insufficiency in the non‐pregnant state, the true incidence of cervical insufficiency remains unknown. The diagnosis of cervical insufficiency is based on a history of painless cervical dilation after the first trimester with subsequent expulsion of the pregnancy in the second trimester, typically before 24 weeks of gestation, without contractions or labor and in the absence of other clear pathology (e.g., bleeding, infection, or ruptured membranes).

One prospective study aimed to determine the value of a cervical length measurement at 11–14 weeks in predicting the risk of a MTL occurring between 16 and 24 weeks; 11 (0.4%) women miscarried between 16 and 24 weeks, whereas 2825 delivered after 34 weeks. CL was significantly shorter (Mann–Whitney *U*‐test, *P* = 0.001) in women who had a MTL in comparison to those who delivered after 34 weeks (median CL 28 mm vs 32 mm, respectively).^20^ First trimester cervical length was predictive of an MTL (AUC = 0.7838, *P <* 0.001).

Meta‐analysis data have shown that surgical treatment for cervical intraepithelial neoplasia (including loop electrosurgical excision procedure) is associated with a significantly increased risk of pregnancy loss in the second trimester.^21^ MTL was assessed in eight studies (*n* = 16 558 women). In this pooled analysis, MTL risk was higher for treated than for untreated women (1.6% and 0.4%, relative risk [RR] 2.60, 95% CI 1.45–4.67).

Emerging evidence has also shown an association between MTL and women who had cesarean section in labor, particularly at full dilatation (FDCS), in a previous term birth. Disruption and scarring near or within cervical tissue might result in cervical weakness in future pregnancies. A cohort study compared the outcomes of pregnant women with a preterm birth in their second pregnancy and either a prior FDCS or a prior term vaginal birth for their first. The main outcome for the study was gestational age at birth and birth <30 weeks in the next (third) pregnancy. Sixty‐six women were included in the analysis, of which 29 women had delivered with a previous history of FDCS. The results appeared to show that the women with a prior history of term FDCS had a threefold increased relative risk of recurrent MTL or preterm birth compared with those women with a history of vaginal births (12/29 vs 5/37, *P =* 0.02, Fisher's exact test, RR 3.06, 95% CI 1.22–7.71).^22^

A cohort of 600 patients with a history of dilation and evacuation (D&E) for MTL was followed into subsequent pregnancies. Of 96 pregnancies identified, 77 resulted in term deliveries. The authors concluded that second‐trimester D&E did not increase the risk of MTL or spontaneous preterm birth.^23^

##### 4.2.2.2 Congenital uterine anomalies

There is a considerable body of evidence describing congenital uterine anomalies (CUA) and early pregnancy loss rates; however, only a minority of studies differentiate between first trimester and MTL. It is important to note that owing to improvements in diagnostic methods, the incidence of CUA is showing an apparent increase, due to previously undiagnosed cases now being ascertained.

A systematic review with meta‐analysis, published in 2014, has estimated the prevalence of CUA to be 5.5% (95% CI 3.5%–8.5%) in unselected women, 8.0% (95% CI 5.3%–12%) in infertile women, 13.3% (95% CI 8.9%–20.0%) in women with recurrent early pregnancy loss, and 24.5% (95% CI 18.3–32.8) in women with infertility and early pregnancy loss. The risk of sporadic MTL was not significantly increased in women with didelphys (RR 1.71, 95% CI 0.63–4.59; four studies) and unicornuate uteri (RR 2.27, 95% CI 0.64–7.96; four studies) versus normal controls. However, women with septate (RR 2.95, 95% CI 1.51–5.77; five studies) and bicornuate uteri (RR 2.90, 95% CI 1.56–5.41; four studies) had a significantly increased risk of sporadic MTL versus controls.^24^

A study from 2023, comparing the reproductive outcomes of women with a unicornuate uterus to women without a CUA, did however seem to indicate an increased risk of MTL. A total of 326 women with a diagnosis of a unicornuate uterus were included and compared with 326 controls. When specifically assessing the risk of MTL between 15 and 22 weeks, there was an increased aOR of 5.19, 95% CI 1.47–18.33.^25^ Additionally, within the aforementioned 2014 systematic review, an arcuate uterus was also thought to increase the risk of sporadic MTL (RR 1.98, 95% CI 1.06–3.69; five studies); however, following this, a large prospective study using only gold standard three‐dimensional (3D) ultrasound for diagnosis did not corroborate this finding, with similar pregnancy and live birth rates noted compared to normal controls.^26^

##### 4.2.2.3 Acquired uterine anomalies

###### 4.2.2.3.1 Myomas

Myomas, depending on size and location within the uterus, are thought to have an impact on fertility and reproductive outcomes to varying degrees. Several potential mechanisms by which myomas affect embryo implantation and ongoing pregnancy have been proposed. These mechanisms include chronic endometrial inflammation, abnormal vascularization, increased uterine contractility, and abnormal local endocrine patterns.^27^

A study performed within a recurrent early pregnancy loss population found that women with myomas were at a higher risk of a MTL compared with women with unexplained recurrent early pregnancy loss. The risk was greatest in women with submucosal myomas (*n* = 13/60, 21.7%) compared to intramural/subserosal (*n* = 22/125, 17.6%), compared with (*n* = 52/651, 8%) risk in the unexplained recurrent early pregnancy loss group (*P* < 0.01).^28^

Further, when women underwent a surgical resection of submucosal myomas, this appeared to significantly reduce MTL risk (*n* = 13/60 (21.7%) to *n* = 0/25 (0%); *P* < 0.01). The lack of a control group limited the strength of the study, and in fact, the women in the intramural/subserosal group were found to have an equivalent live birth rate to the unexplained group in the subsequent pregnancy.^28^

###### 4.2.2.3.2 Adenomyosis

The presence of adenomyosis has also been shown to potentially increase the risk of adverse pregnancy outcomes through various pathogenic mechanisms, including disruption of the uterine junctional zone (JZ) and, thus, of uterine peristalsis;^29^ increased intrauterine oxidative stress that might lead to maternal endothelial dysfunction and abnormal placentation,^30^ and an increased inflammatory environment that might alter myometrial decidualization, resulting in an abnormal trophoblastic JZ invasion during pregnancy.^31^

A Japanese retrospective case–control study compared obstetric complications in 49 singleton pregnancy cases in women with known adenomyosis compared to controls without adenomyosis (*n* = 245). Cases were matched by age, parity, and the need for assisted reproductive technology for conception. Women with adenomyosis were significantly more likely to have an MTL (12.2% vs 1.2%, OR: 11.2, 95% CI 2.2–71.2).^32^

###### 4.2.2.3.3 Placenta problems

Placental insufficiency can impair the transfer of nutrients and oxygen, thereby raising the risk of miscarriage. However, it is important to recognize that certain placental characteristics, such as infarction and calcification, might also occur in uncomplicated pregnancies and are not reliable indicators of poor outcomes.

High‐quality prospective cohort evidence of placental factors specific to MTL risk is sparse. One large population‐based prospective study conducted in South Africa followed 7010 singleton pregnancies with 66 late second‐trimester miscarriages (<22 weeks) investigated. When placental histology was available, the most common findings were abruption (either alone or in combination with maternal vascular malperfusion) and acute chorioamnionitis, followed by isolated maternal vascular malperfusion, and less frequently, chronic villitis (including CMV‐associated cases).^33^

Some histological findings might reflect sequelae of an underlying condition rather than representing a direct placental factor contributing to miscarriage risk. Placental inflammation might well be the downstream consequence of chorioamnionitis. Histologic chorioamnionitis on placental exam, demonstrated by inflammatory cell infiltrates in the chorion, amnion, villi, or intervillous space, after MTL suggests infection as an underlying cause, even if there were no overt maternal signs.

In a case–control study of 90 spontaneous second‐trimester losses and 17 controls, Srinivas et al. (2008)^34^ found histologic chorioamnionitis (neutrophil infiltration of the chorion/amnion) in 67% of losses, compared with none of the controls (*P* < 0.001). Importantly, many affected women had no documented clinical diagnosis of chorioamnionitis, indicating that subclinical infection or inflammation could be an under‐recognized contributor to mid‐trimester loss.

Both inherited and acquired thrombophilias can cause micro‐ and macro‐thrombosis within the spiral arteries and intervillous space. Maternal vascular malperfusion (MVM) is the predominant placental pathology in stillbirth, particularly in the early third trimester, but potentially if the malperfusion is severe or occurs early enough, might lead to MTL. MVM, which presents as multiple placental infarcts, distal villous hypoplasia, and decidual arteriopathy, has been observed in patients with Factor V Leiden or antiphospholipid syndrome (APS) who have had a MTL.^35^

One notable pathology is massive perivillous fibrin deposition (MPFD), characterized by extensive fibrin accumulation around chorionic villi. This disruption of placental function can cause fetal growth restriction and miscarriage, with nearly one‐third of affected pregnancies ending in loss. Notably, the recurrence risk can reach up to 18%.^36^

Placental hematomas are associated with adverse pregnancy outcomes. Data regarding the significance of subchorionic hematoma show a variable relationship with pregnancy outcome, but studies demonstrating subchorionic hematoma in the second trimester suggest a stronger relationship with adverse pregnancy outcome than those identified in the third trimester.^37^

An Austrian retrospective case–control study compared pregnancy outcomes of 32 women with an intraplacental hematoma (located in the intervillous cavity of the placenta), 199 women with a retroplacental hematoma, and a control group consisting of 113 age‐matched women with no signs of placental abnormalities. The presence of an intraplacental hematoma had a higher associated rate of MTL than retroplacental hematoma (*P* < 0.01).^38^

#### 4.2.3 Infectious factors

Studies have found that ascending infection from the lower genital tract is implicated in 10%–25% of MTL.^39^ Women with a prior history of MTL are thought to be at increased risk of preterm birth and vice versa.

The risk of infection‐driven MTL appears to be inversely related to gestational age. In a retrospective study of placental histology of 7705 spontaneous births, the prevalence of histological chorioamnionitis was found to be 94.4% in births occurring between 21 and 24 weeks, compared to 40% in births at 25–28 weeks and 10.7% in births at 33–36 weeks.^40^

Chorioamnionitis is a polymicrobial condition. The organisms involved can include anaerobic streptococci, enterococci, coliforms, staphylococci, fusobacterium, mycoplasma, urea plasma, and group B hemolytic streptococci. It is thought that the symptoms of chorioamnionitis are most associated with the ascent of micro‐organisms via the cervix into the uterine cavity. Histological examination of placental tissue in births occurring between 16‐ and 24‐weeks' gestation has shown localized choriodecidual inflammation, generalized chorioamnionitis, intervillositis, and funisitis.^41^

The causative micro‐organisms that are implicated with preterm birth appear to also be linked to MTL. One theory is that the deleterious effects caused by these micro‐organisms, if rapidly progressive, might result in MTL, and if more slowly progressive, could lead to a later viable but extremely preterm birth.^42^

In healthy women, the normal genital tract flora consists for the most part of *Lactobacillus* species bacteria.^41^ Potentially virulent microbes, such as *Gardnerella vaginalis*, can displace lactobacilli as the predominant organisms in the vagina, a condition known as bacterial vaginosis (BV).

Vaginal dysbiosis (with or without symptoms of BV) has been proposed as a mechanism that can induce local inflammation and invasion by infectious agents, potentially increasing pregnancy loss risk. Although this process has been implicated in early pregnancy loss overall, the data available in relation to MTL is ambiguous.^43^

Meta‐analysis data, reviewing all women screened positive for BV either by clinical criteria or by criteria based on gram‐stain findings, were noted to show an increase in the risk of MTL with asymptomatic bacterial vaginosis (OR 6.32, 95% CI 3.65–10.94), as well for preterm birth and maternal infection.^43^

A 2013 Cochrane review aimed to assess the effects of antibiotic treatment of BV in pregnancy. The review included 21 trials, totaling 7847 women diagnosed with bacterial vaginosis or intermediate vaginal flora. Antibiotic treatment was found to reduce the risk of MTL (RR 0.20; 95% CI 0.05–0.76; two trials, 1270 women, fixed effect, *I* ^2^ = 0%).^44^

The largest randomized controlled trial that has been conducted since this review, however, appeared to show that the treatment of BV did not decrease MTL risk. The PREMEVA trial screened 84 530 pregnant women before 14 weeks gestation. Of the 5630 women noted to have BV, 2869 were randomly assigned to receiving a single course of clindamycin, a triple course of clindamycin, or placebo. The primary outcome was a composite of MTL (16–21 weeks) or spontaneous very preterm birth (22–32 weeks), which was assessed in all patients with birth data (modified intention to treat). The primary outcome occurred in 22 (1.2%) of 1904 participants receiving clindamycin and 10 (1.0%) of 956 participants receiving placebo (RR 1.10, 95% CI 0.53–2.32; *P =* 0.82).^45^

Women with a history of MTL (from 16 to 21 weeks + 6 days) or preterm birth (from 22 to 36 weeks + 6 days) were eligible for inclusion into a separate high‐risk subtrial and were randomly assigned (1:1) to either single‐course or triple‐course clindamycin. For these 236 high‐risk pregnancies, the primary outcome occurred in five (4.4%) participants in the triple‐course clindamycin group and 8 (6.0%) participants in the single‐course clindamycin group (RR 0.67, 95% CI 0.23–2.00; *P =* 0.47).

A more recent clinical trial published in 2023, evaluating a screen and treat policy for BV compared to standard practice, also appeared to show no difference in pregnancy loss <22 weeks in the low‐risk population.^46^

Pertaining to the impact of BV on MTL loss, as the diagnostic criteria for dysbiosis remain unclear, with the lack of standardized screening and detection methods, it remains unclear whether a screen and treat should be implemented.

##### 4.2.3.1 Urinary tract infections

The relationship of urinary tract infections (UTIs) with MTL is difficult to determine due to limited data. However, it is important to acknowledge that there is data on the association between UTI and PTB, and this might be relevant in the context of MTL. UTIs are classified based on the site of infection: lower urinary tract (urethritis cystitis) or upper urinary tract (pyelonephritis).

A number of observational studies have demonstrated the relationship between maternal symptomatic UTI and the risk of sPTB and low birth weight.^47^, ^48^ Preterm deliveries are significantly higher in mothers with pyelonephritis. However, there is substantial heterogeneity between the studies and possible bias, which makes it difficult to establish the overall contribution of UTIs to preterm birth.^49^ GBS bacteriuria is considered a marker for genital tract colonization with GBS, which poses a significant risk of PPROM, sPTB, and early‐onset severe neonatal infection.^50^, ^51^

##### 4.2.3.2 Asymptomatic bacteriuria

Asymptomatic bacteriuria (ASB) is the presence of one or more species of bacteria growing in the urine at specified quantitative counts (≥105 colony‐forming units [CFU]/mL or ≥108 CFU/L), irrespective of the presence of pyuria, in the absence of signs or symptoms attributable to UTIs. It can occur in 2%–10% of pregnancies;^45^ however, the effect on perinatal outcomes is unclear.^52^, ^53^

Available evidence does not demonstrate a direct association between ASB and MTL. Most trials and observational studies on ASB in pregnancy focus on the benefits of early detection and treatment in preventing pyelonephritis, with mixed and low‐certainty evidence for reducing preterm birth and none reporting MTL as a specific outcome. Registry and surveillance data likewise record infection as a broad category in relation to MTL, without specifying ASB. Although ascending infection from the urinary tract is considered a recognized pathway to MTL, there remains no high‐quality, gestation‐specific evidence that ASB alone, when identified and treated, alters MTL risk in unselected obstetric populations.

##### 4.2.3.3 Other maternal infections

In severe maternal infection, such as with influenza, HIV, dengue fever, and malaria, the maternal immune response might result in MTL instead of a direct placental infection effect. Pathogens such as *Plasmodium* parasites and Dengue fever's flavivirus have been known to be detected in fetal tissue and placenta.^54^

Specifically, regarding viral infections, a case–control study was performed to determine if placental viral infection was associated with spontaneous MTL.^55^ Eighty‐four patients with spontaneous MTL (15–23.6 weeks) were included in multivariable model analysis. The study found that the presence of any human papilloma virus was significantly associated with MTL loss (OR = 5.64, CI 1.35, 23.56), as was cytomegalovirus (CMV) (OR = 5.04, CI 1.03–24.45). When histological chorioamnionitis was added to the multivariate model, however, only CMV remained a statistically significant predictor of MTL (OR = 4.62, CI = 1.02–20.89).

Parvovirus B19 (B19V) is also able to cross the placenta and result in a fetal infection. This might lead to severe fetal anemia (similar to pregnancies affected by red cell alloimmunization), hydrops fetalis, early pregnancy loss, or intrauterine fetal death. A systematic review and meta‐analysis, exploring the outcomes of 611 fetuses affected by B19V, found that the occurrence of hydrops was associated with a higher risk of pregnancy loss both up to 20 weeks (OR 11.5; 95% CI 2.7–49.7) and after 20 weeks or up to 28 days postpartum (OR 4.2; 95% CI 1.6–11.0).^56^

#### 4.2.4 Thrombophilia

##### 4.2.4.1 Acquired

Antiphospholipid syndrome is characterized by the association between adverse pregnancy or vascular thrombosis outcomes and the presence of antiphospholipid antibodies, anticardiolipin antibodies, or anti‐beta‐2‐glycoprotein‐I antibodies. One or more morphologically normal fetal losses after the tenth week of gestation is considered adequate to meet the criteria for an adverse pregnancy outcome in APS.

The hypothesis behind APS and pregnancy loss is largely based on the development of a prothrombotic environment. APS‐associated MTL is thought to be related to poor vascularization, leading to impaired placental function, probably secondary to inflammation and thrombosis. Abnormal histologic findings in the spiral arteries have included narrowing, intimal thickening, acute atherosis, and fibrinoid necrosis.^57^

Meta‐analysis data, including a total of 25 studies examining APS and recurrent fetal loss, showed that the presence of lupus anticoagulant (OR 7.79, 95% CI 2.30–26.45) and IgM anticardiolipin antibodies (OR 5.61, 95% CI 1.26–25.03) was associated with late recurrent fetal loss. IgG anticardiolipin antibodies, when combining all titers, were associated with both early (OR 3.56, 95% CI 1.48–8.59) and late recurrent fetal loss (OR 3.57, 95% CI 2.26–5.65).^58^

##### 4.2.4.2 Inherited

Over the past several decades, progress has been made in the identification and understanding of inherited hypercoagulable disorders that promote thrombosis, collectively termed inherited thrombophilia.

Meta‐analysis data appears to show that the significance of the association between inherited thrombophilias and fetal loss depends largely on the type of thrombophilia and timing of fetal loss. It is thought that there is a more consistent association between MTL and inherited thrombophilias, with the presumed mechanism being thrombosis of the uteroplacental circulation. Of 31 studies included, Factor V Leiden was associated with early (OR 2.01, 95% CI 1.13–3.58) and late (OR 7.83, 95% CI 2.83–21.67) recurrent fetal loss and late non‐recurrent fetal loss (OR 3.26, 95% 1.82–5.83). Prothrombin G20210A mutation was associated with early recurrent (OR 2.56, 1.04–6.29) and late non‐recurrent (OR 2.30, 95% CI 1.09–4.87) fetal loss. Protein S deficiency was associated with recurrent fetal loss (OR 14.72, 95% CI 0.99–218.01) and late non‐recurrent fetal loss (OR 7.39, 95% CI 1.28–42.63).^59^

#### 4.2.5 Genetic factors

##### 4.2.5.1 Fetal chromosomal anomalies

A literature review of causes of stillbirth found that the chromosomal causes for pregnancy loss reduced from approximately 50% in the first trimester to approximately 5%–15% in the third trimester; an inference can be made that the rate of second trimester chromosomal cause is likely to be between these ranges.^60^ One study estimated a 25% chance of a chromosomal cause in MTL, which included aneuploidy (trisomy and monosomy) and polyploidy (triploidy and tetraploidy).^61^ Clinical overviews based on cytogenic cohorts have shown that trisomies 13/18/21 and monsomy X are the most common abnormalities observed.^62^

#### 4.2.6 Endocrine factors

Systemic maternal endocrine disorders such as diabetes mellitus and thyroid disease have been associated with early pregnancy loss.

##### 4.2.6.1 Thyroid

Studies have shown that overt hypothyroidism is associated with a substantial risk of early pregnancy loss.^63^ There is controversy as to whether subclinical hypothyroidism (SCH) has the same effect. In an Irish prevalence study from a single pregnancy loss clinic, reviewing results from 262 women, undiagnosed overt or subclinical hypothyroidism was found more often in women with MTL compared with those with recurrent early pregnancy loss (14.6% vs 7.1%).^64^

A systematic review and meta‐analysis sought to evaluate the relationship between SCH and the risk of early pregnancy loss before 20 weeks of pregnancy. The American Thyroid Association recommended criteria of TSH >2.5 mIU/L in early pregnancy for defining SCH was used in five studies (*n* = 25 158 women); compared with euthyroid pregnant women (*n* = 22 528), patients with non‐treated SCH (*n* = 2630) had a higher prevalence of early pregnancy loss (RR = 2.07, 95% CI 1.70–2.53, *P* < 0.01).^65^

##### 4.2.6.2 Polycystic ovary syndrome

Polycystic ovary syndrome (PCOS) has been linked to an overall increased risk of early pregnancy loss, but the exact mechanism remains unclear.^66^ A retrospective cohort study conducted in China, including 15 210 pregnancies following IVF treatment, noted that PCOS was found to be an independent risk factor for MTL (aOR 1.58, 95% CI 1.28–1.96). The odds ratio was adjusted for the type and duration of infertility, previous spontaneous pregnancy loss (gestation not specified), and stage of the embryo.^6^

A post‐hoc analysis was conducted on three prospective, double‐blind studies involving 791 pregnant women with PCOS, who were randomized to receive either metformin or placebo from the first trimester until delivery. Women diagnosed with gestational diabetes (GDM) at baseline in the first trimester had a statistically significantly higher risk of late miscarriage compared to those without (GDM) (*P* < 0.01).^67^

#### 4.2.7 Male factors

Although there appears to be an association between sperm DNA fragmentation and overall risk of early pregnancy loss, there is currently a lack of available evidence in relation to male factors contributing specifically to mid‐trimester loss.^68^

### 4.3 Long‐term consequences of mid‐trimester pregnancy loss

The long‐term consequences following MTL are yet to be well studied. It is important to note that studies from women with recurrent pregnancy losses have demonstrated an increased risk of maternal cardiovascular risk and venous thrombosis.^69^

There is a strong association with psychological conditions including anxiety, depression, and suicide with pregnancy loss. A multicenter, prospective cohort study of 537 women following a first trimester pregnancy loss found that 9 months after the loss, 18% of women met the criteria for post‐traumatic stress (PTS), 17% for moderate of severe anxiety, and 6% for moderate or severe depression.^70^ A systematic review and meta‐analysis of PTS and PTS disorder reported that losses at progressively later gestational ages were associated with higher levels of PTS/PTSD.^71^

## 5 TRIAGE AND ASSESSMENT OF WOMEN WITH SYMPTOMS OF THREATENED MID‐TRIMESTER PREGNANCY LOSS

### 5.1 Initial assessment (history‐taking)

As with any assessment, this must start with a comprehensive history of the presenting complaint. This is important to evaluate possible etiology (fetal, placental, uterocervical, or infective) and therefore target investigations and management.

Women with threatened MTL might present with subtle symptoms such as increased vaginal discharge or pressure in the vagina. In some circumstances, the woman might be asymptomatic and the diagnosis of MTL is made during a routine ultrasound examination.

Extra attention must be given to the details of any previous MTL or extreme preterm birth, particularly if the birth occurred at home, in a different hospital, or was not investigated.

It is important to ensure that women are listened to and their fears are acknowledged. This is particularly relevant if they have experienced a previous loss. If a woman feels her symptoms are significant, this needs to be factored into subsequent management decisions. These factors are especially important for women from minority ethnic groups whom we know report difficulties in having their symptoms or concerns taken seriously.

Key questions include:

- Previous pregnancy losses (with the gestational age of occurrence), extreme preterm births or stillbirths (with the gestational age of occurrence), antecedent rupture of membranes, and whether the fetus was born showing signs of life prior to 22 weeks.
- The presence or absence of vaginal bleeding, the presence and duration of pain, contractions or backache, fevers, history of antibiotic therapy, previous (or current) cerclage or progesterone use, and a history of multiple attendances with recurrent symptoms in pregnancy.^39^

A review of the notes, scan reports, and investigation results from the current and previous pregnancies should be undertaken, including scans undertaken elsewhere or in early pregnancy units.

A template clinical assessment document incorporating a structured clinical history sheet is provided in Table S1 (Table 1).

**TABLE 1:** Initial triage and assessment of women with threatened mid‐trimester pregnancy loss.

Recommendations	Quality of evidence^a^	Strength of recommendation
Women should be assessed using a standardized approach in a maternity setting. When this is not possible, clear communication, pathways and transition of care from gynecology to obstetrics is necessary.	⊕○○○	Strong
Assessment should include an examination, tailored to the presenting symptoms, as well as abdominal and speculum examination. This should be performed by someone with experience in visualizing the cervix.	⊕○○○	Strong
A local standard operating procedure might facilitate this assessment being performed by any member of the clinical care team with the necessary skills (including nurses or midwifes).	⊕○○○	Strong
Women with confirmed or threatened MTL should be offered care in a side room and if they attended alone an offer to call someone to attend to support them	⊕○○○	Strong
The primary team should be the maternity team with anesthetic support, as necessary.	⊕○○○	Strong
Women should have all clinically appropriate analgesia options available to them.	⊕○○○	Strong
Best practice would be for the woman's care to be coordinated by a named midwife with support from the bereavement team if there is a confirmed MTL.	⊕○○○	Strong

Abbreviation: MTL, Midtrimester pregnancy loss.

^a^ Quality of evidence has been assessed using the GRADE system throughout. ⊕⊕⊕⊕ = quality is high, the authors have a lot of confidence that the true effect is similar to the estimated effect, ⊕⊕⊕o = quality is moderate, ⊕⊕oo = quality is low, ⊕ooo = quality is very low.

### 5.2 Examination and bedside tests

Maternal observations should be recorded at admission (heart rate, blood pressure, temperature, respiratory rate, and oxygen saturations). This should be recorded on a Modified Early Obstetric Warning Score (MEOWS) chart if available and escalated or repeated according to score or concerns.

An abdominal examination should be performed to assess for any palpable contractions or tenderness.

A perineal examination should be done with collection of a low vaginal swab (LVS) for GBS.

Vaginal speculum examination is required to assess for bleeding, cervical dilatation, bulging or rupture of membranes and to collect samples for microbiological culture. If a cervical polyp is seen, best practice is to avoid removal during the pregnancy.

If the cervix is not clearly visualized and in the absence of ruptured membranes, a gentle vaginal examination might be considered. However, it might be more appropriate to escalate to a more senior colleague or consider transvaginal ultrasound assessment of cervical length. If the cervix is dilated, particular attention should be paid to avoid rupturing the membranes. Sterile precautions should be adopted prior to any examination where PPROM is suspected. Women with confirmed PPROM are at risk of ascending infection and sepsis. Therefore, speculum or digital examinations should be kept to a minimum and undertaken by experienced practitioners with sterile precautions. In women experiencing vaginal spotting, further investigations might still be needed regardless of the presence or absence of an ectropion.

Transvaginal ultrasound scanning provides additional information to speculum examination and should be performed at the bedside when there is a skilled provider available to do so. Transvaginal scanning is superior to transabdominal scanning for cervical length assessment. It is important to note that a “long closed cervix” on speculum cannot rule out funneling at the internal os. See Section 6.2 for further detail regarding the role of ultrasound (Table 2).

**TABLE 2:** Examination of women with symptoms of threatened mid‐trimester pregnancy loss.

Recommendations	Quality of evidence^a^	Strength of recommendation
A full set of observations should be recorded at admission (heart rate, blood pressure, temperature, respiratory rate and oxygen saturations). This should be recorded on a MEOWS chart and escalated or repeated according to score.	⊕○○○	Strong
High vigilance should be kept for sepsis and chorioamnionitis.	⊕⊕⊕○	Strong
A speculum examination should be performed by someone with experience in visualizing the cervix.	⊕○○○	Strong

Abbreviation: MEOWS, Modified early obstetric warning score.

^a^ Quality of evidence has been assessed using the GRADE system throughout. ⊕⊕⊕o = quality is moderate, ⊕⊕oo = quality is low, ⊕ooo = quality is very low.

## 6 INVESTIGATIONS

### 6.1 Laboratory tests

It is important to explain to women how they should collect an uncontaminated midstream specimen of urine (MSU). A high vaginal swab (HVS) should be collected if a speculum examination is performed. An LVS should be sent in all cases as this is more sensitive for GBS detection. It is important to put the indication on any microbiology request as the microbiologist might give different advice depending on the clinical situation (at a minimum, it is necessary to specify threatened pregnancy loss and gestation). Ideally, urine and swabs should be collected before giving any antibiotics. If samples are collected after giving antibiotics, there is a chance of a false negative result. In this situation, the timing and type of antibiotic given should be documented on the microbiology request.

Testing for sexually transmitted infections is important as most infections are asymptomatic. This might be focused on at‐risk groups (e.g., under 25‐year‐olds) or routine practice depending on local prevalence. Testing, treatment, and follow‐up should follow local guidelines. This might involve allied services such as genitourinary medicine.

The sensitivity and specificity of leukocytosis and raised C‐reactive protein in the prediction of chorioamnionitis is poor. Thus, these investigations should be used in conjunction with clinical features when diagnosing chorioamnionitis (Table 3).^5^

**TABLE 3:** Recommended laboratory tests for women with symptoms of threatened mid‐trimester pregnancy loss.

Blood test recommendations	Quality of evidence^a^	Strength of recommendation
A full blood count should be taken.	⊕○○○	Strong
A C‐reactive protein should be considered—may be useful in women with ruptured membranes or suspected infection.	⊕⊕○○	Strong
A group and save should be taken if significant bleeding is present, or not booked for antenatal care, as well as a Kleihauer if Rhesus negative.	⊕⊕○○	Strong
A septic screen should be taken if maternal infection is suspected.	⊕⊕⊕○	Strong

Microbiology/other test recommendations	Quality of evidence^a^	Strength of recommendation
A MSU for MC&S should be taken. The MSU should be sent off urgently, as a delay might increase the chance of a false negative. A negative urinalysis does not exclude a UTI. Urine should still be sent for MC&S in all cases and possible MTL or PPROM clearly noted on the request. This might prompt extended cultures, so it is important to include this information.	⊕⊕○○	Strong
A low vaginal swab for GBS should be sent and a high vaginal swab might be considered (for other vaginal infections), as well as a swab for chlamydia (NAAT).	⊕⊕○○	Strong
Samples should be collected before giving any antibiotics. Antibiotics should be prioritized in unwell women if there is a delay in obtaining samples. If samples are collected after giving antibiotics, a negative result will have a higher chance of being falsely negative.	⊕○○○	Strong
Women with symptoms of vaginal discharge or previous loss/vaginal infection should be screened for bacterial vaginosis.	⊕○○○	Strong
If a woman reports a gush of fluid from the vagina but there is no clinical evidence of liquor on sterile speculum, insulin‐like growth factor binding protein 1 test or placental alpha‐macroglobulin 1 test of vaginal fluid might be considered, but false positive rates are high. Interpretation of results is to be guided by ongoing symptoms and liquor volume on scan. PPROM is unlikely if no ongoing fluid loss and liquor volume is normal on scan over several weeks. It is not recommended to use these tests in isolation.	⊕○○○	Conditional

Abbreviation: GBS, group B Streptococcus; MSU, Midstream urine; MTL, midtrimester pregnancy loss; MC&S, mocroscopy, culture and sensitivity; NAAT, nucleic acid amplification test; PPROM, preterm prelabor rupture of membranes; UTI, urinary tract infection.

^a^ Quality of evidence has been assessed using the GRADE system throughout. ⊕⊕⊕o = quality is moderate, ⊕⊕oo = quality is low, ⊕ooo = quality is very low..

### 6.2 Ultrasound investigations

#### 6.2.1 Transabdominal (TA) and transvaginal (TV) ultrasound scan

It is recommended that women receive both transabdominal TA and transvaginal ultrasound if possible, as bedside tests (see Section 5).

A scan to assess fetal viability should be performed as soon as possible and is recommended as part of the initial assessment to guide onward management.

Transvaginal cervical length measurement is recommended but it is critical that this is performed and interpreted by someone with adequate training. Transvaginal scans provide more accurate measurement; transabdominal scans might not view or accurately assess the cervix resulting in inappropriate management. Cervical length measurements should follow the ISUOG guidelines.

An assessment of liquor volume might be indicated as oligohydramnios is associated with a poorer prognosis, particularly in PPROM. However, a normal liquor volume does not exclude PPROM. A placental location scan should also be performed to assess for the presence of a subchorionic hematoma or low‐lying placenta. This will assist onward care planning, particularly in the case of confirmed MTL.

A detailed ultrasound should be performed by fetal medicine specialists if there are concerns regarding fetal anomaly or liquor volume in the absence of PPROM (Table 4).

**TABLE 4:** Recommended ultrasound investigations for women with symptoms of threatened or confirmed mid‐ trimester loss.

Ultrasound investigations recommendations	Quality of evidence^a^	Strength of recommendation
A viability check should be performed as soon as possible.	⊕○○○	Strong
At least within 24 h, a scan should be performed to assess for cervical length, liquor volume and placental localization.	⊕○○○	Strong
At least within 48 h, a scan should be arranged to assess for a subchorionic hematoma.	⊕○○○	Conditional
A detailed ultrasound should be performed by fetal medicine specialists if there are concerns regarding fetal anomaly, or liquor volume in the absence of PPROM.	⊕○○○	Conditional

Abbreviation: PPROM, Preterm prelabour rupture of membranes.

^a^ Quality of evidence has been assessed using the GRADE system throughout. ⊕ooo = quality is very low..

## 7 MANAGEMENT OF THREATENED MID‐TRIMESTER PREGNANCY LOSS

Section 7.1 details the basic standard care that all women with threatened MTL should receive. Sections 7.4, 7.10 detail specific additional steps that might be required depending on the suspected cause.

### 7.1 Threatened mid‐trimester pregnancy loss

Women with threatened mid‐trimester pregnancy loss should be managed primarily by the maternity team. Threatened mid‐trimester pregnancy loss should never be classed as an “inevitable miscarriage”. Management should target the likely cause with support from the preterm prevention team. It might be necessary to modify the management plan depending on clinical circumstances. It is recommended that a named midwife/doctor should coordinate the woman's care and ensure follow‐up (whatever the pregnancy outcome). Women should be counseled based on gestation, clinical condition, and likely prognosis. There should be shared decision‐making with the woman where the individual's risk, as well as the potential benefits, harms and practicalities of proposed interventions are discussed.

The QUiPP app is a decision support tool that combines medical history and cervical length to give an individualized score for the risk of imminent birth. This app provides a risk score currently validated from 18 weeks' gestation in asymptomatic high‐risk women but only from 23 weeks in those with symptoms. It is, therefore, not applicable in this cohort of women with symptoms between 14 + 0 and 21 + 6 weeks but might be relevant in low‐resource settings if the guideline is applied up to 28 weeks.

Emergency cerclage and progesterone might be considered from 16 weeks (see below).^72^, ^73^ If birth is expected, parents should be counseled that their baby will sadly not survive. Neonatal resuscitation is not usually offered at less than 22 weeks' gestation and, therefore, counseling would not be expected to involve the neonatal team.^4^

Conversations with parents should be clearly documented and care taken to ensure that the agreed management plan is handed over between professionals and staff shifts. Decisions and management should be regularly reviewed in conjunction with the parents. Plans might be reconsidered if there is a change in clinical circumstances or parental wishes.^4^

#### 7.1.1 If the baby is born (at any gestation) with signs of life

There have been reports of fetuses born with signs of life as early as 13 weeks. These signs include spontaneous breathing, presence of a heartbeat, pulsation of the umbilical cord, or definite movement of voluntary muscles. Fleeting reflex activity observed only in the first minute after birth is not classified as a sign of life. Doctors should review at the earliest opportunity, and in the event of subsequent death a neonatal death certificate might be issued if applicable and according to local regulations.

### 7.2 Progesterone

There are no studies reporting on the effectiveness of progesterone for threatened mid‐trimester pregnancy loss, so its use cannot be routinely recommended. Progesterone's effectiveness for reducing first trimester loss^73^ and preterm birth^74^ is acknowledged, so it might be that in selected groups its use is beneficial in the mid‐trimester too. This reflects the potential overlap in etiology between MTL and first‐trimester miscarriage on the one hand and MTL and preterm birth on the other. Progesterone can, therefore, be considered at acute presentation, for example if a short cervix is suspected but transvaginal cervical length measurement is not immediately available, or after specialist assessment for a recognized indication such as short cervical length.^72^

Vaginal progesterone is a micronized natural formulation and is, therefore, the preparation that is recommended. The dose recommended is in line with that used for preterm prevention and, therefore, 200 mg at night. The importance of good hygiene should be discussed with the woman to ensure that the risk of infection by contamination is minimized. For women who do not tolerate vaginal progesterone, rectal administration might be considered. If should be noted that clinical trial data to support this route of administration is lacking, and advice to avoid the risk of contamination from the rectum to the vagina is crucial.

### 7.3 Cerclage

The FIGO (2021) good practice recommendations on cervical cerclage^75^ suggest cervical cerclage for women with a history of spontaneous second trimester loss or preterm birth if they have a short cervix (<25 mm in singletons and potentially <15 mm in twins). Cerclage is not routinely recommended for women incidentally found to have a short cervix with no other risk factors for preterm birth. Consensus opinion supports offering cervical cerclage for women presenting with threatened MTL if cervical insufficiency is suspected to be the cause. If cervical dilatation and exposed membranes are diagnosed, emergency cervical cerclage could be considered after senior clinical review and counseling. Contraindications to cervical cerclage include significant bleeding, infection, rupture of membranes or pain, as these might indicate that cervical insufficiency is not the cause and, therefore, raise the maternal risks without the benefit of prolongation of pregnancy.

### 7.4 Management of urinary tract infections

An MSU should be sent to assess for asymptomatic bacteriuria. If the woman is symptomatic of UTI, then an MSU should still be sent, with treatment commenced while waiting for the results. Choice of antibiotic should be based on the pattern of local UTI causative organisms and resistance profiles. If the subsequent culture does not confirm an infection, then antibiotics can be stopped.

If a UTI is caused by Group B streptococcus (GBS), this should be treated. If GBS growth is noted but is below the standard UTI treatment threshold, it is not known what the optimal management should be; but, in general, if reported by microbiology, treatment should be considered (especially if symptomatic or PPROM).

Anytime an infection is diagnosed and treated, a test of cure should be performed (a repeat microbiology sample 48–72 h after completion of antibiotics).

### 7.5 Management of microbial dysbiosis

Clindamycin can be considered in women with abnormal vaginal flora.^76^

Bacterial vaginosis should be treated with vaginal metronidazole or clindamycin to reduce the risk of mid‐trimester pregnancy loss or PPROM (RR 0.85; 95% CI 0.62 to 1.17; five trials, 4088 women; *T* ^2^ = 0.06, *I* ^2^ = 49%).^44^, ^58^, ^77^

There is unclear evidence regarding the benefit of treating GBS identified on an LVS. This should be recorded in maternity care notes, and if pregnancy progresses, intrapartum antibiotics should be offered. In the case of MTL, intrapartum antibiotics could be considered, but further evidence is needed.

Ureaplasma was found to be the most frequently detected infectious agent in the vaginal cultures from pregnant women, regardless of gestational age. Additional studies are required to confirm that diagnosis and treatment according to laboratory results of vaginal infections with *Ureaplasma urealyticum* or *Mycoplasma hominis* during the first trimester of pregnancy could prevent premature birth, MTL, or chorioamnionitis.^77^

The decision to prescribe any antibiotic should consider that antibiotics might worsen dysbiosis and can be associated with harm. The use of antibiotics for women in preterm labor with no clinical evidence of infection did not improve perinatal outcome, and follow‐up of the children at 7 years showed an increase in functional impairment in those whose mothers were given erythromycin.^78^

### 7.6 Preterm pre‐labor rupture of membranes

In the situation of extreme PPROM, counseling regarding onward management should include discussions about the risk of significant maternal and fetal morbidity and mortality. Options can include expectant management with vigilance for sepsis or termination of pregnancy.

For women <20 weeks' gestation, there is no evidence to guide clinical decision‐making. For this reason, the provision of antibiotics <20 weeks should be an individualized clinical decision, taking into consideration patients' preferences. If the decision is to give antibiotics, women with preterm pre‐labor rupture of membranes can be treated with oral erythromycin 250 mg QDS for a maximum of 10 days or until the woman is in established labor (whichever is sooner), as per NICE guidelines (consider penicillin‐based treatment if allergic to erythromycin).^72^ Once this decision has been made, we do not advocate new or additional courses of prophylactic antibiotics throughout the pregnancy. The aim is to reduce the chance of ascending infection and allow the pregnancy to reach a viable gestation.

### 7.7 Management of subchorionic hematoma

The presence of a subchorionic hematoma on ultrasound scan should be noted in women presenting with bleeding or pain in mid‐trimester. The size should be documented to determine whether there is a massive subchorionic hematoma (defined as >1 cm thickness occupying >50% of the placenta).

Progesterone pessaries might be considered but should not be offered routinely. In the presence of bleeding in a woman taking low dose aspirin and/or LMWH counseling should include an individualized risk–benefit discussion regarding continuation of anticoagulation. If anticoagulation is stopped, then timing for review and conditions for restarting should be agreed and documented.

Women should be counseled regarding the increased risk of pregnancy complications such as preterm birth and fetal growth restriction. For these reasons, they should be offered follow‐up in a specialist clinic to assess cervical length and fetal growth, as per local guidelines. If available, uterine artery Doppler and estimated fetal weight at 20–24 weeks' gestation might help further assess risk such that if both are normal, serial growth scans can be delayed to 32 weeks. If uterine artery Doppler is unavailable, serial assessment of fetal growth from 28 weeks' gestation is appropriate.^79^

### 7.8 Management of antepartum hemorrhage

This should be managed according to the amount of bleeding and the condition of the woman.

In the event of major bleeding, this should be managed by stabilizing the woman, expediting birth or considering termination of pregnancy (with early recourse to surgical management).^80^

For mild or moderate bleeding, women should be admitted for observation for a minimum of 24 h and anti‐D given if indicated. Due to the risk of intrauterine growth restriction with recurrent bleeding, serial scans should be arranged from 28 weeks.^63^

### 7.9 Management of short cervix

It is important that cervical length assessment is performed by appropriately trained practitioners. Management should take account of the full clinical history and tailor treatment to the likely cause. The treatment will most likely be progesterone and/or cerclage (see Sections 7.2 and 7.3) but should recognize that infection might cause a short cervix, and therefore treatment for infection might be needed as a first line. Cervical cerclage is not recommended in the presence of active bleeding or infection.

### 7.10 Multiple pregnancy

Management of threatened mid‐trimester pregnancy loss in multiple pregnancy requires input from multiple pregnancy specialists and is not discussed in detail here. Clinicians should appreciate the complexity and mixed emotions of couples who experience MTL, termination, or selective reduction of one fetus with a surviving twin or higher order multiple. The timing and mode of birth will depend on chronicity, gestation, the position of the fetuses and the well‐being of the surviving baby or babies. Fetal medicine advice should be sought (Table 5).^81^

**TABLE 5:** General management of threatened mid‐trimester pregnancy loss.

Recommendations	Quality of evidence^a^	Strength of recommendation
Management should be targeted to the suspected cause.	⊕○○○	Strong
Threatened MTL should not be automatically treated as an inevitable loss.	⊕○○○	Strong
Care pathways should exist for management between 14 + 0 and 21 + 6 weeks' gestation. Ideally, this care should be provided by the obstetric team. When this is not possible clear communication, pathways and transition of care from gynecology to obstetrics is necessary.	⊕○○○	Strong
Women with evidence of chorioamnionitis need review by a senior clinician and should have broad spectrum intravenous antibiotics started without delay. There should be consultant involvement in the decisions for onward management.	⊕ ⊕ ○○	Strong
There is no direct evidence for the use of progesterone for women with threatened MTL. Women may be considered for vaginal progesterone treatment in the acute setting if a short cervix is suspected until transvaginal cervical length measurement is obtained. The decision for continuing progesterone, for instance if a short cervix is identified, should involve preterm prevention specialists.	⊕ ⊕ ○○	Conditional
If a short cervix is diagnosed cervical cerclage can be considered if there is no ongoing pain or bleeding. The decision should involve preterm prevention specialists.	⊕ ⊕ ○○	Conditional

Abbreviation: MTL, Midtrimester pregnancy loss.

^a^ Quality of evidence has been assessed using the GRADE system throughout. ⊕⊕oo = quality is low, ⊕ooo = quality is very low.

## 8 MANAGEMENT OF IN‐UTERO DEATH, CONFIRMED MID‐TRIMESTER PREGNANCY LOSS

If fetal demise has occurred and the woman is hemodynamically stable, it is best to ensure she has a side room and a partner, or another person of her choice, with her. One‐to‐one compassionate care should be provided with bereavement and chaplaincy support, as appropriate. The induction‐to‐birth interval should be minimized to prevent systemic infection developing but guided by the wishes of the woman and her clinical condition. Some women will want to start the induction process as soon as possible; others might wish to go home, have time to process the diagnosis, feelings and thoughts, and see family members first.

If the woman had been feeling fetal movements before diagnosis, then the possibility of passive movements should be discussed with her, as this explanation might be important to her.

Women should be counseled regarding expectant, medical, and surgical management. An urgent care (emergency) pathway should be followed for women bleeding excessively or those who are systemically unwell. A less urgent (elective) pathway can be followed for women with no or mild bleeding.

The woman and her partner should be included in the discussions about management options and their wishes should be respected. Written information leaflets (in appropriate languages) should be offered where available.

Vaginal birth is recommended at gestations under 24 weeks. The recommendation regarding mode of birth in complex cases should be made by a consultant. In the case of placenta previa, vaginal birth might still be recommended but availability of blood products and facilities for immediate recourse to operative birth is needed in the event of hemorrhage. Rarely, a surgical birth (dilatation and extraction in a specialist center, or hysterotomy) might be required for maternal hemodynamic instability, a stenosed cervix, a morbidly adherent placenta, to preserve a trans‐abdominal cerclage (TAC), or for maternal choice. It is possible to remove a TAC laparoscopically or via a posterior colpotomy to enable women to deliver vaginally.^82^, ^83^

Analgesia is particularly important for women who labor. All clinically appropriate options should be available, including regional anesthesia and woman‐controlled analgesia. Assessment for maternal coagulopathy and sepsis might be required prior to regional anesthesia.

Care should ideally be provided in a “bereavement suite”. A bereavement suite is a dedicated room within or near a labor ward but away from the sounds of laboring women or newborns. These rooms were originally set up for the purpose of birthing stillborn babies. Women should be given the choice, if possible, of where they would prefer to be cared for, especially at the earlier gestation range (14–16 weeks). Women should be cared for by staff trained in bereavement care. The UK National Bereavement Care Pathway (NBCP) outlines recommendations for providing excellent care to parents after loss.^84^

### 8.1 Expectant management

More than 85% of women with an intrauterine fetal death (IUFD) deliver spontaneously within 3 weeks of diagnosis.^85^ If the woman is physically well, with a closed cervix and intact membranes, the risks related to expectant management are low. However, there is a 10% chance of maternal coagulopathy 4 weeks from the date of fetal death.^85^ Expectant management is not recommended in cases or regions where rapid access to health care and follow‐up is not guaranteed.

If birth is delayed by more than 48 h, a full blood count (FBC) and clotting screen should be repeated twice weekly.^86^ should be advised that with expectant management, the appearance of baby might deteriorate over time. All women should be given a 24‐h contact number and advised to return if they have significant abdominal pain, vaginal bleeding, concerns about their well‐being, or if they change their mind and wish to proceed with expediting the birth.

Where PPROM is also present with IUFD, the risk of chorioamnionitis and maternal sepsis should inform counseling. Expediting birth is recommended as expectant management is associated with higher chances of chorioamnionitis, postpartum hemorrhage, admission to intensive care, and hysterectomy.^87^, ^88^

### 8.2 Medical management of confirmed mid‐trimester pregnancy loss

**FIGURE 1:**
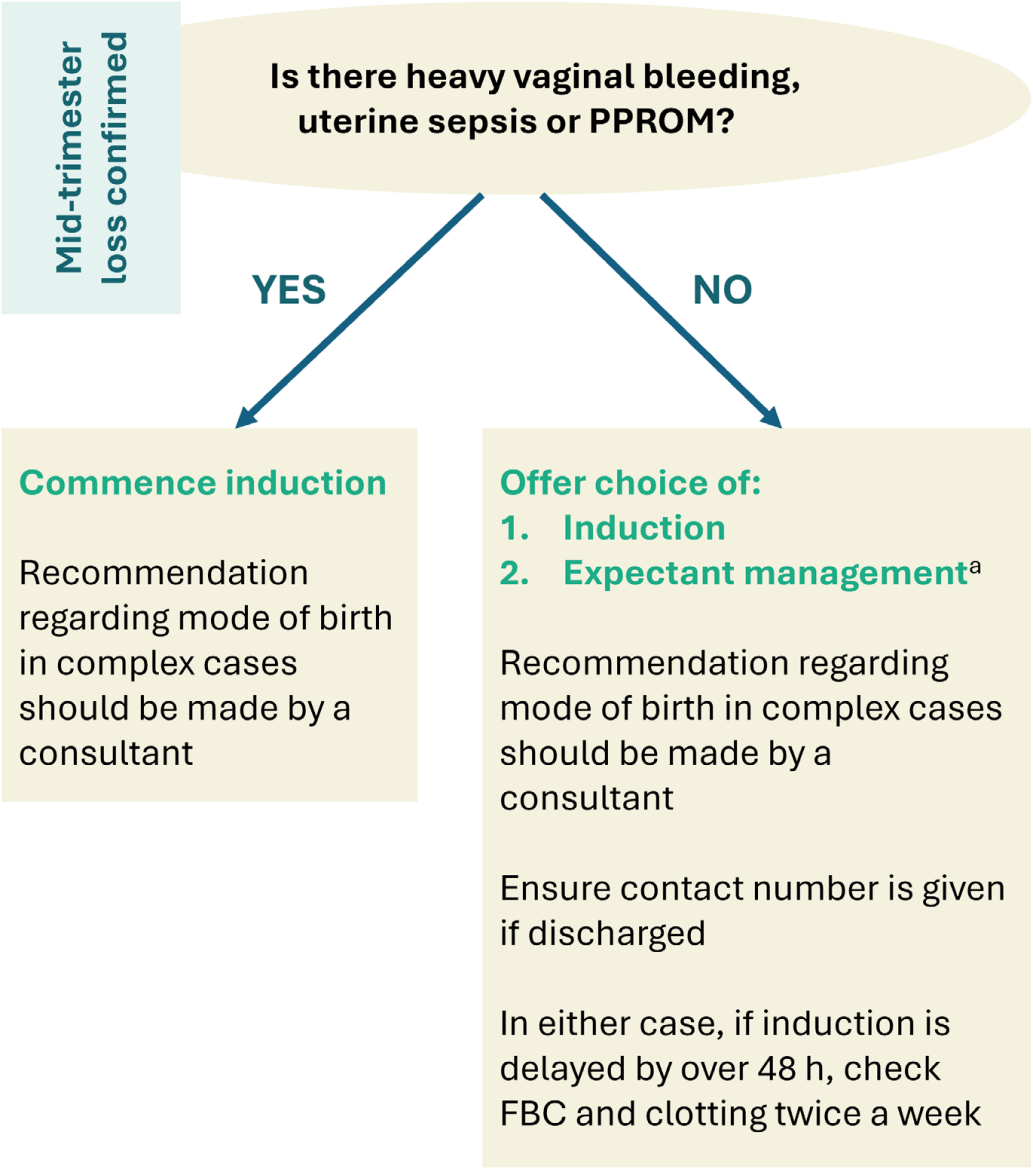
Decision tool for the medical management of confirmed mid‐trimester pregnancy loss. PPROM, preterm pre‐labor rupture of membranes. ^a^Expectant management is not recommended in cases or regions where rapid access to healthcare and follow‐up is not guaranteed.

Decision tool for the medical management of confirmed mid‐trimester loss (see Figure 1).

The use of a combination of mifepristone (an anti‐progestogenic steroid) and misoprostol (a synthetic prostaglandin) is recommended as the first‐line pharmacological intervention for induction in MTL.^88^, ^89^ Initial management with a single dose of 200 mg oral mifepristone is recommended. The usual interval between mifepristone and misoprostol is 1–2 days, although this can be shortened if clinically needed. A 2‐day interval between mifepristone and misoprostol administration might decrease the duration of misoprostol administration,^89^ the time in hospital, the need for surgical evacuation, and the use of hospital resources.^90^ We recognize that there are different regimens used globally and that the agents used for cervical ripening might be dictated by local resources (Table 6).

**TABLE 6:** Induction regimen for women with confirmed mid‐trimester pregnancy loss.

Induction recommendations		Quality of evidence^a^	Strength of recommendation
Pre‐induction	Mifepristone 200 mg should be used orally once only.	⊕⊕⊕○	Strong
Induction	Misoprostol 400 μg can be used via the buccal, sublingual or vaginal route, 3 hourly until birth of the fetus (91). It can be used with unscarred and scarred uterus (but with caution with the latter see Section 8.2.1). Vaginal use may be associated with fewer side effects.		

^a^ Quality of evidence has been assessed using the GRADE system throughout. ⊕⊕⊕o = quality is moderate.

Misoprostol effects are dose dependent and include cervical softening and dilation and uterine contractions, as well as side effects such as nausea, vomiting, diarrhea, fever, and chills. Although fever is a side effect of misoprostol, the woman should still be closely monitored clinically for any signs of infection.

The required amount of misoprostol decreases with increasing gestational age and if the fetus has died in utero.^91^ Due to the lower risk of side effects, the vaginal route is preferable in the absence of PPROM, bleeding or infection.^92^

Most women experience birth of the fetus with up to five doses of misoprostol. If birth is not achieved after five doses, management should be discussed with a consultant. Further doses of misoprostol might achieve birth so doses above the regime can continue or be restarted after a 12‐h rest.^88^ Additional doses are not associated with safety concerns.^93^

If membranes are ruptured, oxytocin infusion can be considered as a method of induction.

Extra care must be taken to minimize the risk of infection. Postpartum hemorrhage and retained placenta need to be anticipated and should be actively managed. In the event of a delay of more than 30 min in the delivery of the placenta after the birth of the fetus, an additional dose of misoprostol can be given. This is recommended to be 400–800 μg depending on the level of bleeding.^88^

#### 8.2.1 Medical management of a scarred uterus

The risk of uterine rupture with misoprostol is small but might be increased in women with MTL and a history of cesarean section or other uterine scars.^89^ Healthcare professionals should follow local protocols and be vigilant to clinical features suggestive of uterine scar dehiscence or rupture, such as scar pain or bleeding. Misoprostol can be safely used for induction of labor in women with a single previous lower‐segment cesarean section. Local protocols might recommend lower doses of misoprostol or increased time between doses, particularly with atypical scars (e.g., classical cesarean or open myomectomy) or more than one previous uterine scar, although this is not mandated.

### 8.3 Surgical management

Surgical uterine evacuation might be necessary for women with persistent excessive bleeding, hemodynamic instability, evidence of infected retained tissue, or suspected gestational trophoblastic disease or for maternal choice.

Surgical management should be carried out under ultrasound guidance. The pregnant uterus is vulnerable to perforation (risk 1:1000). Other complications include hemorrhage (requiring blood transfusion 0–3:1000), infection (40:1000), and cervical trauma (<1:1000).^94^ Active measures such as tranexamic acid, oxytocin, and misoprostol should be used or be available to manage postpartum hemorrhage.

Women should be informed that there is a higher incidence of retained pregnancy tissue with medical or expectant management of MTL compared to first trimester pregnancy loss. A low threshold for offering surgical evacuation should be considered if the placenta or membranes appear incomplete or if the woman experiences excessive bleeding. In cases of suspected retained pregnancy tissue, an ultrasound assessment should be arranged to help with the diagnosis. Women with large amounts of retained tissue are at higher risk of postpartum hemorrhage (Table 7).^95^

**TABLE 7:** Management of confirmed mid‐trimester pregnancy loss (fetal death).

Recommendations	Quality of evidence^a^	Strength of recommendation
Women should be admitted to a bereavement room, where their emotional and practical needs can be considered without compromising safety.	⊕○○○	Conditional
Care should be provided by an experienced midwife or nurse. Best practice would be to provide continuity of care. The duty consultant should be made aware of the admission. All care‐providing staff should be trained in bereavement care.	⊕○○○	Conditional
Women should be counseled regarding expectant, medical and surgical options. Vaginal birth is preferred for birth less than 24 weeks' gestation (even with placenta previa).	⊕○○○	Strong
An urgent care (emergency) pathway should be followed for women bleeding excessively or those who are systemically unwell. A less urgent (elective) pathway can be followed for women with no or mild bleeding.	⊕○○○	Strong
All clinically appropriate pain relief options should be available including regional anesthesia and woman‐controlled anesthesia.	⊕○○○	Strong
For women who opt for expectant management, if birth is delayed by more than 48 h, a full blood count and clotting screen should be done and repeated twice weekly	⊕○○○	Conditional
A combination of mifepristone and misoprostol is recommended for induction in mid‐trimester pregnancy loss.	⊕⊕⊕○	Strong (Conditional where mifepristone is not available)
Surgical uterine evacuation may be necessary for women with persistent excessive bleeding, hemodynamic instability, evidence of infected retained tissue, suspected gestational trophoblastic disease, or for maternal choice.	⊕⊕○○	Strong
Surgical management should ideally be carried out under ultrasound guidance.	⊕⊕○○	Strong
Active measures such as tranexamic acid, oxytocin and misoprostol must be available to prevent or manage post‐partum hemorrhage.	⊕○○○	Strong

^a^ Quality of evidence has been assessed using the GRADE system throughout. ⊕⊕⊕o = quality is moderate, ⊕⊕oo = quality is low, ⊕ooo = quality is very low.

## 9 POSTNATAL CARE AFTER MID‐TRIMESTER PREGNANCY LOSS

### 9.1 Psychological support

Women should be cared for in a separate bereavement room (i.e. in a different environment from women with live babies) by trained staff. Continuity of care should be provided. The partner (or a friend or family member) should be allowed to stay and have open visiting.

The grief of the woman and her partner is commonly profound and long‐standing following MTL. It affects their physical and emotional well‐being and might have negative effects on their extended family. Several studies have found that posttraumatic stress (PTS) or post‐traumatic stress disorder (PTSD) can occur following an MTL.^96^, ^97^, ^98^ A systematic review of 48 studies found that PTS is more prevalent than PTSD; however, the prevalence of both conditions decreased over time.^71^ These conditions were reported more in women and with a higher gestational age. A qualitative study found that PTSD can persist for at least 3 months.^98^ Factors that might be protective for some women, such as seeing and holding the fetus, might be associated with distress in other women, so options should be explained to women and families and their choices respected. Healthcare professionals should be aware of the risk of PTSD and remain vigilant to identify those at risk and offer appropriate onward care.

A narrative systematic review of six studies focusing on parents' (the woman and her partner) experiences and perceived holistic needs following MTL identified three main themes: communication and information‐giving; feelings post‐event; and the impact of support provision. Literature about the experience of MTL is scarce, with what was found reporting a lack of compassionate and individually tailored psychological follow‐up care for parents following MTL. Further research is required to inform and develop this area of maternity care provision.^96^

The woman and her partner often complain of feeling isolated once they return home; therefore, it is essential that the community team supports them and their family once they are discharged home. Extra care and support are needed in the long term and especially during and after all subsequent pregnancies. Women with pre‐existing psychiatric illness or little social support are particularly vulnerable to psychological morbidity following an MTL.^97^

The practical implications for postnatal health care include provision of clear and tailored information, creating opportunities for parents to say goodbye, giving sufficient attention to their emotional well‐being, and ensuring a respectful hospital environment.^98^

A national survey of MTL care in the UK found that the advantage of managing women in the delivery suite is to utilize the expertise of trained midwives who can offer emotional support to mothers who have just lost a baby. There are also benefits to being close to an operating room and having ready access to anesthetic support. The disadvantage of the delivery suite is that women might find it distressing to be in the immediate vicinity of pregnant mothers and newborns.^99^

Staff should have an honest conversation with the woman and her partner and gently explain what their baby might look like after birth, and they should always be offered the opportunity to see or hold their baby, regardless of the gestation. Some might wish to see and hold their baby immediately after birth, others might prefer to wait, and some will decline. Their decision should be respected. The woman and her partner should be offered the use of a cooling cot if available.

The woman and her partner should be aware that the sex of the infant might not be easily identified at this gestation. Hence, in cases of uncertainty, the fetal sex should not be assigned. Confirmation of sex might be available through cytogenetics or post‐mortem examination. They might decide on a neutral name for their baby as these results might take several weeks to be reported. If the woman and her partner have chosen a name for their baby, this should be recorded in the medical notes with the parents' consent.

The woman and her partner should be directed to bereavement support services. They should be provided with appropriate literature and contact phone numbers. They should have the option of photographs and memories being made. The woman and her partner should be given information regarding burial and cremation and be allowed to make their own choice in keeping with their religious and cultural beliefs.

### 9.2 Physical care

Non‐sensitized Rhesus (Rh) negative women should receive prophylactic anti‐D immunoglobulin.^84^ Women should be assessed for risk of thromboprophylaxis according to national or local guidelines.

Before discharge, the woman should be reviewed by a specialist midwife, nurse, or obstetrician. Women should be provided with printed information on supportive care (from support groups as well as local pregnancy loss services and charities) and contact numbers. It is important that any existing antenatal appointments should be canceled so that reminders are not inadvertently sent. The primary care doctor should be notified of the MTL.

Suppression of lactation with 1 mg cabergoline can be offered to women who reached 18 weeks or more of gestation. Contraindications to cabergoline include severe pre‐eclampsia and hypertension. A randomized controlled trial found that compared to placebo, cabergoline is effective and well tolerated to prevent breast symptoms after MTL;^100^ however, bromocriptine 2.5 mg twice a day is still an option in low‐resource settings. Contraception should be discussed before discharge.

### 9.3 Transfer of baby

Where facilities exist, parents should be supported to spend as much time as they wish to spend with their baby before transfer to the mortuary. Hospitals should enable this by providing cold cots. Where these facilities do not exist, care should be in line with local practice.

Occasionally, the family might wish to take their baby home. Parents should be made aware that the baby's appearance might deteriorate rapidly. However, the parents' wishes should be supported, taking into account local legal requirements regarding taking the baby home.

The baby should be taken home in an appropriate casket or moses basket. The transport to home must be appropriate (i.e. private and not public transport). The mortuary must be informed if the parents are taking their baby home (Table 8).

**TABLE 8:** Postpartum care of women who have suffered a mid‐trimester pregnancy loss.

Recommendations	Quality of evidence^a^	Strength of recommendation
Parents often report feeling isolated once they return home; therefore, it is essential that the community team offer support after discharge. Extra care and support are needed in the long term and especially during and after subsequent pregnancies.	⊕○○○	Conditional
Couples should be directed to bereavement support services. They should be provided with appropriate literature and contact phone numbers. The option of photographs and memory‐making should be offered. Information regarding burials or cremation should be given and religious or cultural beliefs supported. Where these facilities do not exist, care should be in line with local practice.	⊕○○○	Conditional
Non‐sensitized Rhesus negative women should receive prophylactic anti‐D immunoglobulin.	⊕○○○	Strong
If women want suppression of lactation, cabergoline 1 mg should be offered from 18 weeks of gestation unless there are contraindications such as pre‐eclampsia or severe hypertension.	⊕⊕○○	Strong
Parents should be supported to spend as much time with their baby as they wish before transfer to the mortuary. Cold cots should be used, or where these facilities do not exist, care should be in line with local practice.	⊕○○○	Conditional
Checklists should be used to ensure standardized practice. Care and actions should be recorded.	⊕○○○	Conditional

^a^ Quality of evidence has been assessed using the GRADE system throughout. ⊕⊕oo = quality is low, ⊕ooo = quality is very low.

## 10 POSTNATAL INVESTIGATIONS AND FOLLOW‐UP

### 10.1 Maternal investigations

Postnatal maternal investigations are carried out to assess maternal well‐being and to aid diagnosis of the cause of MTL. These investigations are similar to those carried out after a stillbirth^101^ and should be tailored to the clinical history and examination findings. If the cause of the pregnancy loss is known, for example a short or open cervix, then postnatal investigations should be tailored to this. Investigations are ideally performed at the diagnosis of a mid‐trimester loss (to guide acute management and counseling), with follow‐up testing in the postpartum period if initial workup is inconclusive.

#### 10.1.1 Immediate investigations

Checking the booking FBC report is recommended to exclude severe anemia or hemoglobinopathy. A repeat FBC might be useful if there is excessive bleeding or concerns about maternal infection.^102^ A blood group and antibody screen should be performed to exclude hemolytic disease due to maternal sensitization to red cell antigens. A Kleihauer test should be taken to detect feto‐maternal hemorrhage.

Other tests to be considered include:

- Coagulation and plasma fibrinogen if maternal sepsis, placental abruption, or pre‐eclampsia is suspected or diagnosed.
- Renal and liver function tests might be performed, if abnormalities are suspected, for example, related to severe infection, viral hepatitis, cytomegalovirus, or toxoplasmosis.
- Maternal serology for TORCH screen and parvovirus B19 might be indicated where fetal infection is suspected.
- Maternal urine toxicology should be sent if there is any suggestion of recreational drug use.
- Microbiological tests to be performed if symptoms of infection, to include maternal blood cultures, mid‐stream urine analysis, vaginal/cervical swabs, and placental swabs from the maternal surface.^101^

#### 10.1.2 Further investigations

Where the cause is unknown, and the baby is morphologically normal, investigations for antiphospholipid syndrome (lupus anticoagulant and anticardiolipin antibodies) should be conducted from 6 weeks postnatally. If lupus anticoagulant or anticardiolipin antibodies are positive, they should be repeated after a further 12 weeks. Thyroid function tests (TFTs) (TSH and T4) and thyroid peroxidase antibodies are similarly recommended. There might be a transient thyroiditis following a pregnancy loss, so if TFTs are abnormal just after an MTL, a repeat test is recommended after 2 months. An HbA1c might be useful if risk factors for undiagnosed diabetes are present. Other investigations might be warranted on the basis of the fetal post‐mortem examination (see below).

Ultrasound and hysteroscopy can be useful for the assessment of the uterine cavity after MTL. Hysteroscopy is generally used for assessment of the endometrium and uterine cavity, whereas ultrasound, including 3D scanning, can identify congenital uterine anomalies and be used to examine the myometrium and the adnexa. Ultrasound assessment is especially important if there is a suspicion of a uterine anomaly (e.g., with recurrent MTL). The results from a retrospective observational study compared 3D ultrasound to hysteroscopy, and the concordance between the two was very good with a kappa of 0.83.^103^

### 10.2 Fetal examination

Post‐mortem examination includes a range of procedures, from completely non‐invasive (external examination and imaging), partially or minimally invasive (imaging‐guided autopsy) to full conventional post‐mortem examination.

Whether the parents accept a post‐mortem investigation of the baby or not, it is important that histological testing of the placenta, cord, and membranes is offered. Placental swabs should be taken using aseptic technique for aerobic and anaerobic bacterial cultures. In a large cohort of 5000 cases, placental examination gave a cause of death in around 20% of cases.^104^ Fetal cytogenetic analysis, most commonly chromosome microarray (CMA), should be offered in all cases of MTL. This might be best performed by sampling placental tissue. If CMA shows an abnormality, then parental testing is recommended.

Parents should be given time to consider their options for further testing of their baby. Individual, cultural, and religious beliefs must be respected. The value of post‐mortem in stillbirth is well documented.

External examination is a comprehensive external examination of the baby. It might be helpful in diagnosing obvious fetal structural abnormalities, particularly of the body wall, face, hands, or feet.

Minimally invasive examination: Comprehensive post‐mortem imaging of the baby can now be used to direct whether an invasive postmortem examination or tissue sampling is needed.^105^ Skeletal imaging (e.g., X‐ray) is routinely performed to detect skeletal anomalies, but the yield is low where antenatal imaging is normal and should be reserved for when anomalies are suspected.^106^

Cross‐sectional imaging such as comprehensive postmortem ultrasound or magnetic resonance imaging (MRI) can be offered. A diagnostic accuracy of 70%–80% when compared with invasive postmortem can be achieved.^107^, ^108^ Micro computed tomography and high field MRI can be used but are usually only available in specialist centers.^108^ Tissue biopsy can be performed laparoscopically^109^ or via the umbilicus^110^ if parents do not consent to full autopsy.

Post‐mortem examination: Full conventional autopsy examination is the reference standard for maximum diagnostic information following second trimester loss.

If endomyocardial fibroelastosis or atrioventricular (AV) node calcification is found post‐mortem, maternal anti‐SSA (anti‐Ro) and anti‐SSB (anti‐La) antibodies should be tested. Maternal alloimmune antiplatelet antibodies should be tested for where fetal intracranial hemorrhage is found or fetal thrombocytopenia detected.

Establishing a cause‐and‐effect relationship might be difficult but the use of a careful history to establish the sequence of events and confirm the phenotype of the loss is important. This allows investigations to be tailored to the phenotype. Multidisciplinary team review that includes a pathologist, obstetrician, and neonatologist will offer the best chance of finding a cause.^111^

There should be clarity on who is responsible for following up, reviewing and acting upon the tests results. Parents should be made aware that the overall cause of pregnancy loss remains unexplained in up to half of cases.^7^ Parents should be advised that a follow‐up appointment with a consultant obstetrician, or another appropriate healthcare provider, will be arranged and results of investigations and implications for care in future pregnancies will be discussed. Appointments should take place away from the maternity setting (Table 9).

**TABLE 9:** Postnatal investigations and follow‐up.

Postnatal investigation recommendations	Quality of evidence^a^	Strength of recommendation
Initial (pregnancy baseline) FBC report should be checked to exclude severe anemia or hemoglobinopathy. FBC should be repeated if there is bleeding or infection.	⊕⊕○○	Strong
A blood group, antibody screen and Kleihauer should be taken.	⊕⊕○○	Strong
Other useful blood tests may include coagulation, fibrinogen, and renal and liver function tests, as indicated by the maternal condition (see text)	⊕○○○	Conditional (on clinical presentation)
If the cause of MTL is unknown, the following should be tested for: thyroid function tests (TSH and T4) and thyroid peroxidase antibodies. HbA1c	⊕⊕○○	Conditional (on clinical presentation)
Ultrasound and out‐patient hysteroscopy may be useful for assessment of the uterine cavity	⊕○○○	Conditional (on clinical presentation)

Fetal examination recommendations	Quality of evidence^a^	Strength of recommendations
Fetal cytogenetic analysis should be offered in all cases of MTL. If cytogenetic testing reveals an abnormality, parental testing is recommended.	⊕⊕○○	Strong
Post‐mortem examination may include: External Minimally invasive examination Full autopsy	⊕⊕○○	Conditional (on available resources)
Irrespective of whether the parents accept post‐mortem investigations, histological testing of the placenta, cord, and membranes should be performed.	⊕⊕○○	Conditional (on available resources)
If endomyocardial fibroelastosis or AV node calcification is found at post‐mortem, maternal anti‐SSA (anti‐Ro) and anti‐SSB (anti‐La) antibodies should be tested.	⊕○○○	Conditional (on clinical presentation)
Maternal alloimmune antiplatelet antibodies should be tested where fetal intracranial hemorrhage is found or fetal thrombocytopenia detected.	⊕○○○	Conditional (on clinical presentation)

Follow‐up care recommendations	Quality of evidence^a^	Strength of recommendation
A follow‐up appointment with an appropriate clinician should be arranged to discuss results of investigations and management plan for subsequent pregnancies.	⊕○○○	Strong
The woman and her partner should be counseled that a post‐mortem provides the most information and could affect care in future pregnancies. They should be informed of local timescales and advised that it might take 12 weeks or more to receive the autopsy results. If women opt for external examination only, they should be aware that this is a limited examination.	⊕⊕○○	Conditional (on available resources)
If the woman and her partner have given the baby a name, healthcare professionals should use the baby's name in discussions with the family.	⊕○○○	Strong

Abbreviation: AV, atrioventricular; FBC, Full blood count; HbA1c, haemaglobin A1c; MTL, midtrimester pregnancy loss, TSH, thyroid stimulating hormone; T4, thyroxine.

^a^ Quality of evidence has been assessed using the GRADE system throughout. ⊕⊕oo = quality is low, ⊕ooo = quality is very low..

## 11 RISK OF RECURRENCE AND IMPLICATIONS FOR FUTURE PREGNANCIES

The priorities when planning forward are to provide contraception to allow a choice about interpregnancy interval; to establish the cause in the interval before the next pregnancy; and to develop a plan of care for the subsequent pregnancy based on the likely cause of the MTL.

The overall recurrence rate of MTL is 7%–8% but differs by cause or phenotype, from <5% (in cases of fetal anomaly, multiple gestations, intrauterine fetal death) to 30% (in cases of cervical insufficiency).^7^

A systematic review and meta‐analysis of 10 observational studies reporting on outcomes of 12 004 subsequent pregnancies after MTL generated estimated outcome frequencies for women with a previous MTL as follows: live birth 81% (95% CI: 64–94), early pregnancy (first or second trimester) loss 15% (95% CI: 4–30), and preterm birth 13% [95% CI: 6–23]. The pooled odds ratio for preterm birth in subsequent pregnancy after MTL in case–control studies was OR 4.52 (95% CI: 3.03–6.74). However, as the evidence is limited and of very low certainty, larger, higher quality cohort studies are needed to investigate this potential association (Table 10).^112^

**TABLE 10:** Recurrence risk of mid‐trimester pregnancy loss.

Recommendations	Quality of evidence^a^	Strength of recommendation
Management in subsequent pregnancies should be individualized and care tailored to the likely cause of MTL.	⊕○○○	Strong

Abbreviation: MTL, Midtrimester pregnancy loss.

^a^ Quality of evidence has been assessed using the GRADE system throughout. ⊕⊕⊕o = quality is moderate.

## 12 MANAGEMENT OF PREGNANCIES AFTER MID‐TRIMESTER PREGNANCY LOSS

Management in subsequent pregnancies should be individualized and care tailored to the likely cause of MTL. All women should be booked as high‐risk pregnancies and receive consultant‐led care from a consultant with an interest in preterm birth prevention or pregnancy loss. Their first appointment should be arranged for as soon as possible after their first trimester scan, or earlier if early intervention is indicated.

A study found that parents with a history of prenatal loss, including MTL, experience increased pregnancy‐related stress and anxiety in subsequent pregnancies.^113^ Clinicians should provide the woman and her partner with an opportunity to express their emotional distress and direct them to further psychological support.^113^ Acknowledgment of prior experience in subsequent pregnancy care and encouragement to report any concerns are important (Table 11).

**TABLE 11:** Management of subsequent pregnancies for women who have suffered mid‐trimester pregnancy loss.

Recommendation	Quality of evidence^a^	Strength of recommendation
Women who have an MTL should be advised that there is an increased risk in subsequent recurrence or adverse maternal and neonatal outcomes.	⊕⊕⊕○	Conditional (on reason for the loss)

Abbreviation: MTL, Midtrimester pregnancy loss.

^a^ Quality of evidence has been assessed using the GRADE system throughout. ⊕ooo = quality is very low.

## 13 RESEARCH PRIORITIES

The following questions have been identified as important research questions for mid‐trimester pregnancy loss:

- What is the prevalence of mid‐trimester pregnancy loss (by gestation and cause)?
- What are the sociodemographic risk factors for MTL?
- What is the accuracy and value of cervical length measurement between 14 and 18 weeks in women with symptoms?
- What is the accuracy and value of preterm birth biomarker tests (e.g., fetal fibronectin, Actim Partus, vaginal microbiome) between 14 and 28 weeks in women with symptoms?
- What treatments can reduce mid‐trimester pregnancy loss?
  - Can treatment with progesterone reduce mid‐trimester pregnancy loss for women who present with symptoms?
  - Can treatment with cerclage reduce mid‐trimester pregnancy loss for women who present with symptoms?
- Can non‐surgical management of first/mid‐trimester pregnancy loss or termination of pregnancy reduce future mid‐trimester pregnancy loss when compared with surgical management?
- What is the optimal management of cervical polyps in pregnancy?
- How do infections contribute to mid‐trimester pregnancy loss, and how should they be managed?
- What is the relationship between air pollution exposure and mid‐trimester pregnancy loss?
- What are the consequences of mid‐trimester pregnancy loss in subsequent pregnancies and on maternal physical and mental health?

## AUTHOR CONTRIBUTIONS

This guideline was conceptualized after discussion with the Chair of the FIGO Preterm Birth committee. A consensus group in conjunction with the Tommy's National Centre for Miscarriage Research, Tommy's Maternal and Fetal Health Research Centre, Tommy's National Centre for Preterm Birth Research, and the FIGO Preterm Committee developed the guidance. Evidence synthesis was carried out by CEF, RK, ACare, KV, and JC. The original draft was written by CEF, with all authors contributing to subsequent review and editing. LF and AJD formatted the tables and figures. All authors read and approved the final manuscript.

## FUNDING INFORMATION

This guideline was supported by the Tommy's Charity, who provide funding to the Tommy's National Centre for Miscarriage Research, Tommy's Maternal and Fetal Health Research Centre, and Tommy's National Centre for Preterm Birth Research.

## CONFLICT OF INTEREST STATEMENT

The authors have no conflicts of interest to report.

## Supporting information


Table S1.

## Data Availability

Research data are not shared.
