# Supplementary material for: Triage and care for women with symptoms or diagnosis of pregnancy loss between 14 + 0 and 21 + 6 weeks' gestation
Source: Int J Gynaecol Obstet. 2025 Nov 24;172(1):25–50. doi: 10.1002/ijgo.70621 (PMC12724057; doi:10.1002/ijgo.70621)
Supplement: Supplementary file 1 — Table S1. [file IJGO-172-25-s001.docx]

**Supplementary Table 1** - Standardized history sheet for women with symptoms of MTL

| **Gestation / LMP / dating scan** | | |  |
| --- | --- | --- | --- |
| **Past gynecologic and obstetric history;**   - Cervical smear history, - Cervical surgery, - Previous preterm birth (PTB)/preterm pre-labor rupture of membranes (PPROM) /mid-trimester pregnancy loss (MTL) - Previous caesarean section and dilatation if known - Other associations such as uterine anomaly or antiphospholipid syndrome | | |  |
| **Trigger** | These are important to know before you take a further history as the significance of any symptoms must be viewed in context for that patient | | |
| **Presenting symptoms** (“What is worrying you?”)   - - Spotting/ bleeding | | |  |
| **Trigger** | Any report of vaginal bleeding should raise concern for MTL and prompt careful speculum examination and offer of transvaginal cervical length measurement | | |
| **Presenting symptoms** (“What is worrying you?”)   - - Vaginal discharge/ feeling damp/ loss of mucous plug | | |  |
| **Trigger** | Abnormal discharge/loss of mucous plug is a worrying sign at this gestation and should prompt careful perineal and speculum examination and offer of transvaginal cervical length measurement | | |
| **Presenting symptoms** (“What is worrying you?”)   - - Abdominal pain (site, type, radiation)/ contractions   - Vaginal pressure | | |  |
| **Trigger** | Vaginal pressure is a worrying sign and should prompt careful speculum examination and offer of transvaginal cervical length measurement | | |
| **Presenting symptoms** (“What is worrying you?”)   - - Systemic symptoms- Fever/ malaise   - Systems enquiry –including any change in urinary and bowel symptoms | | |  |
| **Trigger** | | Local or systemic infection may precipitate pregnancy loss so prompt recognition and treatment can prevent this | |
| **Presenting symptoms** (“What is worrying you?”)   - - Trauma – abdominal/ vaginal | | |  |
| **Antenatal care to date:**   - - Booked for pregnancy care?   - Any first trimester complications (include heavy bleeding)?   - Any scans?   - History or UTI in first trimester?   - Asymptomatic bacteriuria at booking?   - Is she under the care of a specialist preterm birth clinic? (+/- on progesterone, cerclage in situ?) | | |  |
| **Rh status;**   - If Rh negative and history of bleeding, assess for anti-D administration | | |  |
| **Recent travel;**   - Covid or flu vaccine, any recent exposure to known infectious disease or sexually transmitted infection such as CMV, chlamydia, parvovirus | | |  |
| **Medical and surgical history** | | |  |
| **Drug history** | | |  |
| **Social history;**   - Support, alcohol, smoking and recreational drugs, domestic violence | | |  |
| **Fetal movements** | | |  |
| **Any other concerns?** | | |  |
